# Exploring the mechanisms of action of the antimicrobial peptide CZS-5 against *Trypanosoma cruzi* epimastigotes: insights from metabolomics and molecular dynamics

**DOI:** 10.1186/s13071-025-06861-5

**Published:** 2025-06-05

**Authors:** Juan Felipe Osorio-Méndez, Daniel Pardo-Rodriguez, Cristian Rocha-Roa, Lily Johana Toro, Laura Muñoz-Tabares, Delia Piedad Recalde-Reyes, Mónica P. Cala

**Affiliations:** 1 Laboratorio de Microbiología y Biología Molecular, Programa de Medicina, Facultad de Ciencias Médicas, Corporación Universitaria Empresarial Alexander von Humboldt, Armenia, Colombia; 2 Metabolomics Core Facility - MetCore, Vice-Presidency for Research, Universidad de los Andes, Bogotá, Colombia; 3 Department of Biology, University of Fribourg, 1700 Fribourg, Switzerland

**Keywords:** *Trypanosoma cruzi*, Epimastigote, Antimicrobial peptides, Cruzioseptins, Mechanism of action, Untargeted metabolomics, Molecular dynamics

## Abstract

**Background:**

Chagas disease, caused by the protozoan parasite *Trypanosoma cruzi*, is a neglected tropical illness affecting an estimated 6–7 million people worldwide. The currently approved drugs have significant limitations, but antimicrobial peptides (AMPs) have emerged as promising therapeutic alternatives. Members of the cruzioseptin family, a group of AMPs derived from the frog *Cruziohyla calcarifer*, have demonstrated anti-*T. cruzi* activity, but their mode of action remains poorly understood. Herein, *T. cruzi* epimastigotes were used to identify active cruzioseptins and investigate their mechanism of action through untargeted metabolomics and molecular dynamics simulations.

**Methods:**

Synthetic versions of three previously unstudied cruzioseptins (CZS-5, CZS-7, and CZS-11) were evaluated for their effects on *T. cruzi* X-1081 epimastigotes via microplate assays with resazurin-based viability measurements. CZS-1, a peptide with known anti-*T. cruzi* activity, was also included. Selectivity was assessed via hemolysis assays on human erythrocytes. To evaluate membrane damage, DNA leakage assays and scanning electron microscopy (SEM) were performed on epimastigotes treated with CZS-5. In addition, the interaction of cruzioseptins with the epimastigote membrane was modeled using molecular dynamics simulations. To explore additional mechanisms of action, a multiplatform metabolomic analysis (HILIC-LC-QTOF-MS and GC-QTOF-MS) was conducted to identify altered metabolites in epimastigotes treated with CZS-5.

**Results:**

Among the tested cruzioseptins, CZS-5 exhibited the highest potency (IC_50_ = 4.7 ± 1.0 µM) and selectivity (SI = 50.3). This peptide induced DNA leakage from epimastigotes and caused surface alterations, suggesting membrane damage. Molecular dynamics simulations indicated that CZS-5 may exert its effects through the formation of toroidal pores in the parasite membrane. Untargeted metabolomic analysis revealed 118 altered metabolites in CZS-5-treated epimastigotes, with significant enrichment of glycerophospholipids (40.7%), supporting the involvement of membrane disruption. In addition, metabolic pathways were affected, suggesting complementary mechanisms of action, including oxidative stress and disruptions in energy metabolism.

**Conclusions:**

CZS-5 was identified as a potent cruzioseptin with multiple potential mechanisms of action in the epimastigotes stage of *T. cruzi*. Further validation is needed in clinically relevant parasite stages to assess its potential as a therapeutic agent.

**Graphical Abstract:**

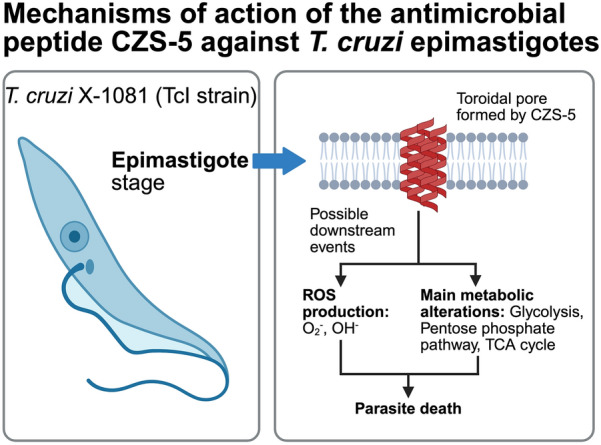

**Supplementary Information:**

The online version contains supplementary material available at 10.1186/s13071-025-06861-5.

## Background

*Trypanosoma cruzi* is the etiological agent of Chagas disease, a neglected tropical illness that affects an estimated 6–7 million people with approximately 12,000 deaths annually, predominantly in Latin America [1]. Approximately 75 million individuals are at risk of contracting the infection. Currently, there are only two approved drugs for treating Chagas disease: benznidazole and nifurtimox [2]. These drugs are generally effective when administered during the acute phase of infection, but their efficacy and safety are significantly compromised in the chronic phase when most patients are diagnosed [3]. Furthermore, parasite isolates exhibit varying susceptibilities to these drugs [4], emphasizing the urgent need for safer and more effective treatments.

AMPs are promising therapeutic candidates because of their potent and broad-spectrum biological activities [5], including strong, selective effects against trypanosomatid parasites [6]. AMPs are small polypeptides that are typically less than 100 amino acids in length and are characterized by their cationic and amphiphilic nature. Produced by the innate immune system of most organisms, these peptides are classified into families on the basis of their source, biological activity, sequence, and physicochemical features [5]. Frogs are a rich source of novel AMPs with therapeutic potential [7–9]. One AMP family is the cruzioseptins, which were isolated from the skin secretions of *Cruziohyla calcarifer*, a frog native to humid tropical forests from eastern Honduras through Panama to northwestern Ecuador [10]. To date, 17 cruzioseptins (CZS-1 to CZS-17) have been identified [10, 11]. These peptides range in length from 20 to 32 residues, are predominantly cationic, and are predicted to adopt an α-helical structure [11]. Synthetic versions of six cruzioseptins (CZS-1 to CZS-4, and CZS-16 and CZS-17) have been evaluated for their biological activities against various microorganisms [10–13]. CZS-1 to CZS-4 and CZS-16 have demonstrated selective activity against both *Leishmania* spp. and *T. cruzi*, with CZS-1 exhibiting the most potent effect against both extracellular and intracellular forms of these parasites [12, 13].

Several antimicrobial peptides with anti-*T. cruzi* activities have been described [6], and multiple modes of action ranging from membrane disruption to targeting intracellular processes have been proposed [6, 14–16]. Detailed knowledge of the mode of action of a given compound or a class of compounds is essential for understanding its therapeutic potential. In this context, untargeted metabolomic analyses and molecular dynamics simulations are key and invaluable tools for achieving this goal [17, 18]. In this study, synthetic versions of four peptides from the cruzioseptin family (CZS-1, CZS-5, CZS-7, and CZS-11) were tested against epimastigotes, the replicative stage of *T. cruzi* found within the insect vector. Mechanistic studies on the most potent and selective identified cruzioseptin (CZS-5) suggest a multifaceted trypanocidal mechanism involving membrane damage, oxidative stress, and other metabolic alterations. To fully establish the therapeutic potential of cruzioseptins, validation of their effect and mechanisms of action is needed in clinically relevant stages of the parasite, such as the trypomastigotes and intracellular amastigotes. The findings reported herein using the epimastigote stage provide a framework for further exploration of the therapeutic potential of cruzioseptins in the treatment of Chagas disease.

## Methods

### Sequence analysis

Amino acid sequences from the 17 reported cruzioseptins were obtained from the literature [10, 11]. Using this information, the following physicochemical features were predicted via the ProtParam (https://web.expasy.org/protparam/, accessed on July 2024) and APD3 (https://aps.unmc.edu/prediction, accessed on July 2024) tools: molecular weight, grand average of hydropathicity (GRAVY), and net charge. The sequences were compared by multiple alignments performed using Clustal Ω (https://www.ebi.ac.uk/Tools/msa/clustalo/). Structural models were constructed in AlphaFold2 [19], and images of the obtained structures were created in Chimera v1.15 [20].

### Peptide synthesis and dilution

All the peptides were obtained by solid-phase chemical synthesis and were subsequently purified via RP-HPLC. The peptides were purchased from Peptide 2.0 (https://www.peptide2.com/) at > 95% purity. Once they arrived, the peptides were diluted in sterile water to 2 mM, dispensed in aliquots, and stored at −80 °C until use.

### Effect of cruzioseptins on epimastigotes

Epimastigotes of *T. cruzi* strains X-1081, Mg, and Ds, belonging to TcI [21], and Y belonging to TcII [22] were grown at 28 °C in liver infusion tryptose (LIT) media [23] supplemented with 10% (*v*/*v*) fetal bovine serum (FBS), 25 mg L^−1^ hemin, and 100 μg mL^−1^ penicillin‒streptomycin. Mid-log epimastigotes were harvested by centrifugation, resuspended in unsupplemented fresh media (*i.e.,* without FBS nor penicillin–streptomycin), and seeded into 96-well microtiter plates (1.5 × 10^6^ parasites per well) containing serial dilutions of the corresponding peptide in a final volume of 100 μL. Each condition was tested in triplicate. Media without parasites and untreated parasites were used as blank and growth controls, respectively. The parasites were incubated at different times with the peptides at 28 °C. Next, resazurin was added to each well at 44 μM and incubated at 28 °C for 2–3 h. Absorbance was measured at 570 and 600 nm using an Epoch microplate reader (BioTek). The same microdilution assay strategy was performed using the antichagasic drug benznidazole (Sigma, 419656), starting at a concentration of 500 µM and incubating for 72 h. Parasite viability was determined via the following formula:

$${\text{Viability }}\left( \% \right) = \left( {{\text{A}}_{{{57}0{\text{ nm}}\_{\text{peptide}}}} {-}{\text{ A}}_{{{57}0{\text{ nm}}\_{\text{blank}}}} } \right) - \left( {{\text{A}}_{{{6}00{\text{ nm}}\_{\text{peptide}}}} {-}{\text{ A}}_{{{6}00{\text{ nm}}\_{\text{blank}}}} } \right)/\left( {{\text{A}}_{{{57}0{\text{ nm}}\_{\text{untreated}}}} {-}{\text{ A}}_{{{57}0{\text{ nm}}\_{\text{blank}}}} } \right) - \left( {{\text{A}}_{{{6}00{\text{ nm}}\_{\text{ untreated}}}} {-}{\text{ A}}_{{{6}00{\text{ nm}}\_{\text{blank}}}} } \right) \, \times { 1}00 \, \%$$

### Hemolysis

The cytotoxicity of the peptides to human erythrocytes was assessed via hemolysis assays. Briefly, 4 mL of venous blood from a healthy volunteer was collected in EDTA tubes and centrifuged at 800 × *g* for 10 min. The resulting erythrocytes were washed three times in phosphate-buffered saline (PBS) and diluted 1:250 in the same solution. This cell suspension was seeded in triplicate into 96-well microtiter plates containing 1:1 serial dilutions of the peptides, with a final volume of 50 μL per well. Untreated erythrocytes and erythrocytes treated with 0.5% (*v*/*v*) Triton X-100 were included as negative and positive controls, respectively. After 18 h at 37 °C, the plates were centrifuged at 800 × *g* for 15 min. Supernatant samples were then used to measure hemoglobin release via spectrophotometry at 540 nm using an Epoch microplate reader (BioTek). The percentage of hemolysis was calculated via the following formula:

$${\text{Hemolysis }}\left( \% \right) = \left( {\left( {{\text{A}}_{{{54}0{\text{ nm}}\_{\text{peptide}}}} {-}{\text{ A}}_{{{54}0{\text{ nm}}\_{\text{blank}}}} } \right){-}\left( {{\text{A}}_{{{54}0{\text{ nm}}\_{\text{untreated}}}} {-}{\text{ A}}_{{{54}0{\text{ nm}}\_{\text{blank}}}} } \right)} \right)/\left( {{\text{A}}_{{{54}0{\text{ nm}}\_{\text{Triton}}}} {-}{\text{ A}}_{{{54}0{\text{ nm}}\_{\text{blank}}}} } \right) \times {1}00\%$$

### Scanning electron microscopy and nucleic acid release assay

To evaluate the effect of CZS-5 on the parasite surface, scanning electron microscopy (SEM) was used. Mid-log epimastigotes of *T. cruzi* X-1081 grown in LIT medium were harvested by centrifugation at 3000 × *g* for 10 min, washed once with PBS, and then transferred to 1.5 mL tubes containing PBS with 5 μM CZS-5. Approximately 5 × 10^6^ parasites were used. After 4 h of incubation at 28 °C, the parasites were harvested by centrifugation as described, resuspended in 2.5% (*w*/*v*) glutaraldehyde, and incubated for 3 h at room temperature. After this, the fixed parasites were centrifuged, washed three times with sterile water, and finally dehydrated with a series of increasing concentrations of ethanol (70%, 90%, and 99.9%). Images of the samples were obtained with a JEOL JSM 6490-LV microscope located at the MicroCore facilities of Universidad de los Andes, Colombia.

To evaluate if the epimastigote’s membrane was damaged by CZS-5, a nucleic acid release assay was performed based on [24]. Briefly, exponentially growing epimastigotes were harvested by centrifugation at 1000 × *g* for 10 min, washed twice with PBS, and then transferred to 1.5 mL tubes containing PBS with various concentrations of CZS-5 in a final volume of 100 μL. Untreated epimastigotes were included as control. After incubation for 2 or 4 h at 28 ℃, samples were centrifuged at 12,000 × *g* for 10 min and the supernatant was measured at 260 nm using an Epoch microplate reader (BioTek).

### Untargeted multiplatform metabolomic analysis

#### Sample preparation

For the untargeted metabolomic analysis, four experimental groups were established: parasites treated with the peptide solubilization vehicle (water), parasites treated with the scrambled peptide (a control peptide with identical amino acid composition but different sequence than the active CZS-5 peptide), and parasites treated with CZS-5 for 2 and 4 h. Epimastigotes of *T. cruzi* X-1081 in the exponential growth phase were exposed to 5 μM of the corresponding peptide (CZS-5 or scrambled CZS-5). The cultures were subsequently centrifuged at 1000 × *g* for 10 min at 4 °C and washed three times with cold PBS. Finally, the cell pellets were stored at −80 °C until analysis at the Metabolomics Core of Universidad de los Andes, Colombia.

Metabolites were extracted by adding 500 µL of cold methanol (−20 °C) to each sample, followed by three cycles of cold sonication (2 min each) to ensure parasite lysis. The samples were subsequently centrifuged at 15,700 × *g* at 4 °C for 20 min, after which the supernatants were collected. Finally, the obtained metabolic extracts were stored at −80 °C for subsequent analysis via liquid chromatography and gas chromatography coupled with high-resolution mass spectrometry, according to the standardized procedures of the MetCore Metabolomics Core [25].

QC samples were prepared by combining equal volumes of the metabolic extract from each sample. The preparation and analysis of the QC samples were then carried out using the same procedures as those described for each analytical platform. To assess the reproducibility of sample preparation and the stability of the analytical platform, multiple QC elutions were performed until the system reached equilibrium. Afterwards, the QC samples were analyzed after every three randomly injected samples.

#### Untargeted metabolomics by GC-QTOF-MS

For gas chromatography/quadrupole time-of-flight mass spectrometry (GC-QTOF-MS) analysis, 100 µL of each metabolic extract was dried in a SpeedVac for 3 h at 35 °C. Then, 10 µL of *O*-methoxyamine in pyridine (15 mg mL^−1^) was added, and the mixture was vortexed for 10 min. The samples were subsequently incubated in the dark for 16 h. After this period, 10 µL of *N*,*O*-bis(trimethylsilyl)trifluoroacetamide with 1% trimethylsilyl chloride was added, and the samples were incubated at 70 °C for 1 h. Following incubation, the samples were cooled to room temperature for 30 min, 100 µL of methyl stearate in heptane (10 mg L^−1^) was added as an internal standard, and the mixture was vortexed for 10 min. The derivatized samples were then immediately analyzed.

For data acquisition, an Agilent Technologies 7890B gas chromatograph coupled to an Agilent Technologies GC/Q-TOF 7250 time-of-flight mass detector was used. The system was equipped with a split/splitless injection port set at 250 °C with a split ratio of 30 and an Agilent Technologies 7693A autosampler. The electron ionization source was operated at 70 eV. Separation was carried out using an Agilent Technologies J&W HP-5MS column (30 m, 0.25 mm, 0.25 µm), with helium as the carrier gas at a constant flow rate of 0.7 mL/min. The oven temperature was programmed to increase from 60 °C (held for 1 min) to 325 °C (held for 10 min). The temperatures of the transfer line, source filament, and quadrupole were maintained at 280 °C, 230 °C, and 150 °C, respectively. Mass spectrometry detection was performed in the range of 50–600 *m/z* at a scanning speed of 5 spectra/min.

#### Untargeted metabolomics by HILIC-LC-QTOF-MS

The samples were analyzed via an Agilent Technologies 1260 liquid chromatography system coupled with a 6545 Q-TOF quadrupole time-of-flight mass spectrometer with electrospray ionization. To perform this analysis, 5 µL of each sample was injected into an ACQUITY UPLC BEH AMIDE column (2.1 mm × 100 mm, 1.7 µm) at 45 °C, with elution using a gradient composed of 10 mM ammonium formate with 0.125% formic acid in water (phase A) and 10 mM ammonium formate with 0.125% formic acid in 95:5 CAN (phase B) at a constant flow rate of 0.4 mL/min. The percentage of phase B was held at 100% for the first 2 min. Subsequently, it gradually decreased to 70% at 7.7 min, 40% at 9.5 min, and 30% at 10.25 min. Thereafter, it increased again to 100% at 12.75 min and remained constant until 17 min. Mass spectrometry detection was performed in negative ESI mode, scanning from 50 to 1100 *m/z*. Two reference masses, *m/z* 112.9856 [C_2_O_2_F_3_(NH_4_)]^−^ and *m/z* 1033.9881 [C_18_H_18_O_6_N_3_P_3_F_24_ + trifluoroacetic acid)-H]^−^, were used for mass correction throughout the analysis.

#### Data processing and analysis

The Agilent MassHunter Profinder program was used to deconvolute, align, and integrate the LC‒MS data with recursive and molecular feature extraction techniques applied. The Agilent MassHunter Unknowns Analysis application, as well as the Fiehn and NIST libraries, were used to deconvolute the GC‒MS data and identify the metabolites. The retention times were then aligned with the Agilent Mass Profiler Professional software, and the results were exported to the Agilent MassHunter Quantitative program for data integration. Both the GC‒MS and LC‒MS data were manually inspected. The data were analyzed for presence and reproducibility, retaining only metabolites that were consistently present or absent in 100% of the samples within the same group and had a coefficient of variance of less than 20% in the QC samples.

To identify molecular features with statistically significant differences among the four groups (CTR, scrambled CZS-5, CZS-5 for 2 h, and CZS-5 for 4 h), univariate and multivariate statistical analyses were performed. For the univariate analysis, *P*-values were determined using nonparametric testing (Mann‒Whitney *U* test). The multivariate analyses used unsupervised principal component analysis (PCA) to observe the sample distribution and supervised orthogonal partial least squares discriminant analysis (OPLS-DA) and partial least squares discriminant analysis (PLS-DA) models to identify the molecular features responsible for group separation. These univariate and multivariate analyses were conducted via the MetaboAnalyst 6.0 server. Statistically significant molecular characteristics were selected using at least one of the following criteria: (1) univariate: *P* < 0.05; and (2) multivariate: VIP > 1.

#### Annotation of statistically significant molecular features

The metabolite annotation during the GC‒MS analysis was identified using the Fiehn version 2013 libraries, the MassHunter Personal Compound Database, and Library Manager Software B.08.00. Multiple metrics were employed to annotate significant characteristics identified via liquid chromatography. These included verifying retention times, estimating the probability of adduct formation, and comparing high-resolution masses to database records via the CEU Mass Mediator server (http://ceumass.eps.uspceu.es, accessed on 7 May 2024). Theoretical formulations were generated using isotope distributions. MS/MS data were compared with spectra from MS-DIAL 4.80 (http://prime.psc.riken.jp/compms/msdial/main.html), Lipid Annotator software version 10.0, and the GNPS server. Manual interpretation of the MS/MS spectra was also carried out.

The identification of compounds through GC analysis was performed by comparing the mass spectrum and FAMES retention index with those reported in the Fiehn GC‒MS Metabolomics RTL (Retention Time Locked) Library 2013 [26]. The Metabolomics Standards Initiative recommendations were used to assign identification levels to each platform [27].

### Coarse-grained molecular dynamics simulations

#### Initial configuration setup

The atomistic structure of each peptide, as predicted using AlphaFold2, was subjected to an energy minimization step until the force on any atom was less than 100 kJ mol^−1^ nm^−1^. For this purpose, the AMBER99SB-ILDN force field [28] and the Steepest descent algorithm were used. The stereochemical quality of the initial and minimized structures was assessed via Ramachandran plots generated by MolProbity [29].

To study the potential membrane pore formation by cruzioseptins, we selected a peptide/lipid ratio of ~0.03 (25 peptide/~820 lipids), as leakage has already been observed in liposomes at ratios similar to antimicrobial peptides [30–32]. Initially, 25 copies oriented perpendicularly to the membrane plane (*i.e.,* transmembrane) were randomly generated using the *gmx_insert-molecules* tool. This configuration was subsequently converted to the Martini CG model using the script Martinize2 [33]. Peptides with high (CZS-5), medium (CZS-11), and low (CZS-1) in vitro activity against *T. cruzi* were used for simulations. On the basis of proteomics data [10], the C-terminal of CZS-1 was capped with an amide group, and for CZS-5 and CZS-11, no capping was used.

Each set of 25 peptides was embedded into a plasma membrane-like bilayer composed of 31% phosphatidylcholine, 54% phosphatidylethanolamine, 4% phosphatidylinositol, and 11% cardiolipin. This membrane represents a model of the plasma membrane from *T. cruzi* in its epimastigote stage [34]. The membrane–peptide configurations were built and solvated using the Insane script [35]. Na^+^ and Cl^−^ martini beads were added to each system to neutralize the charges and reach a concentration of ~0.15 M NaCl. Coarse-grained molecular dynamics (CG-MD) simulations were carried out using Gromacs 2021 software [36] and the Martini 3 force field [37]. For this purpose, the solvated membrane–peptide systems were subjected to two energy minimization stages of 25,000 steps each. This was followed by seven equilibration stages (two under NVT conditions and five under NPT conditions), where the positional restraints on the protein backbone and the lipid phosphate beads were gradually reduced. Then, a production stage was carried out for 20 μs using a time step of 20 fs. The temperature (310 K) and pressure (1 bar) were maintained using the V-rescale thermostat [38] and the Parrinello–Rahman barostat [39], respectively. Three independent replicates were performed for each system. Water and phosphate bead density profiles along the *Z*-axis were calculated via the *gmx_density* tool. Peptide orientation was assessed by measuring the angle between the membrane normal vector and a vector defined by two backbone beads representing each peptide: Phe2 and Leu20 in the case of CZS-1, Phe2 and Lys27 in the case of CZS-5, and Phe2 and Asn26 in the case of CZS-11. The radial distribution functions were computed using the *gmx_rdf* tool. For these calculations, a single bead per peptide copy was used; this bead corresponded to the central/middle backbone bead of the helical structure (Lys11, Ala16, and Gly14, for CZS-1, CZS-5, and CZS-11, respectively). Thus, 25 beads were used per case.

## Results

### In silico characterization of cruzioseptins

**Fig. 1:**
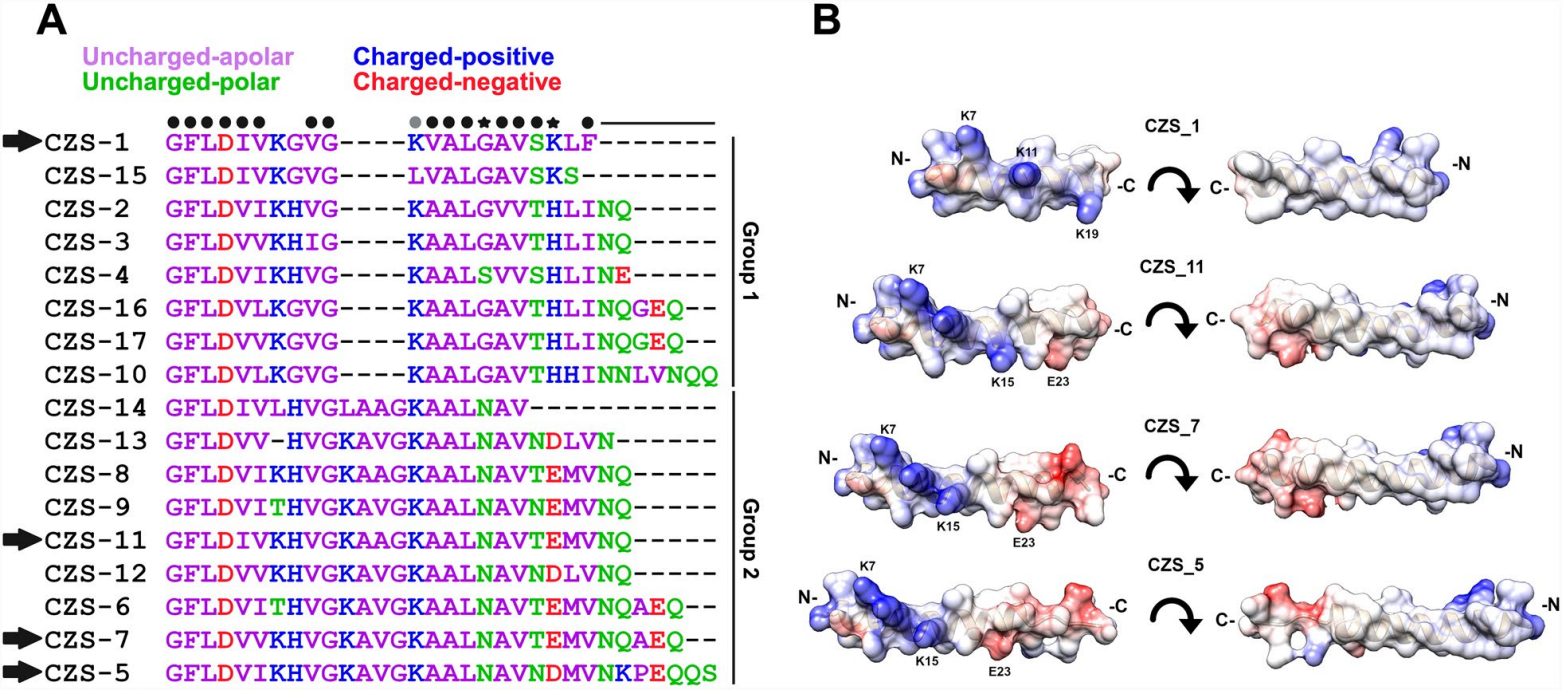
Sequence analysis of peptides from the cruzioseptin family. **A** Multiple sequence alignment of peptides separated into groups 1 and 2. Above the alignment, black and gray circles indicate identical or highly conserved positions, and star positions with nonconservative substitutions that are distinctive for groups 1 and 2. C-terminal extensions are indicated by a horizontal line. Arrows point to the peptides tested in this work. **B** Predicted secondary structure of the tested cruzioseptins, showing their amphipathic nature, with basic and acidic surfaces (blue and red) on one face of the peptide and nonpolar surfaces (white) on the opposite face. C-terminal extensions of cruzioseptins are rich in acidic residues

Cruzioseptins are short, cationic, hydrophobic peptides ranging from 20 to 32 amino acids in length with a high degree of sequence conservation (Fig. 1A). Compared with those of CZS-1, 15 positions across all the cruzioseptins are either identical or exhibit only conservative substitutions. Among these 15 conserved positions, 13 correspond to hydrophobic residues, whereas the remaining two are acidic or polar. These peptides can be classified into two distinct groups on the basis of a four amino acid insertion present in group 2 (Fig. 1A). This insertion is located between residues 10 and 11 of group 1 peptides, and except for CZS-14, this insertion has a highly conserved KA[AV]G motif. Two nonconservative substitutions further distinguish the groups: except for CZS-4, at positions 15 and 19 in group 1, there are Gly and basic (Lys or His) residues, respectively. In contrast, the corresponding positions in group 2 sequences are occupied by Asn and acidic (Asp or Glu) residues, two nonconservative substitutions. In addition, many cruzioseptins also possess C-terminal extensions of up to seven residues, which reduce the overall hydrophobicity and net charge.

Four cruzioseptins were selected for testing against *T. cruzi* epimastigotes (Fig. 1A). CZS-1, a group 1 peptide, was chosen for its previously demonstrated anti-*T. cruzi* and anti-*Leishmania* activities [12, 13]. The other three peptides (CZS-5, CZS-7, and CZS-11) belong to group 2, peptides with no available experimental data. Importantly, they possess key features typical of antimicrobial peptides: (1) a positive net charge (Table 1), and (2) a predicted amphipathic α-helical secondary structure (Fig. 1B). In addition, compared with CZS-1, these latter peptides possess C-terminal extensions ranging from 2 to 7 amino acids in length.

**Table 1:** Predicted physicochemical properties of the tested cruzioseptins

Peptide	Sequence	Length (aa)	Molecular weight (Da)	Net charge	GRAVY
CZS-1	GFLDIVKGVGKVALGAVSKLF	21	2118	+3.00	1.16
CZS-11	GFLDIVKHVGKAAGKAALNAVTEMVNQ	27	2782	+1.25	0.31
CZS-7	GFLDVVKHVGKAVGKAALNAVTEMVNQAEQ	30	3124	+0.25	0.17
CZS-5	GFLDVIKHVGKAVGKAALNAVNDMVNKPEQQS	32	3378	+1.25	−0.17

The peptides are ordered according to their predicted molecular weight

GRAVY, grand average of hydropathicity

### Cruzioseptins selectively kill epimastigotes

**Fig. 2:**
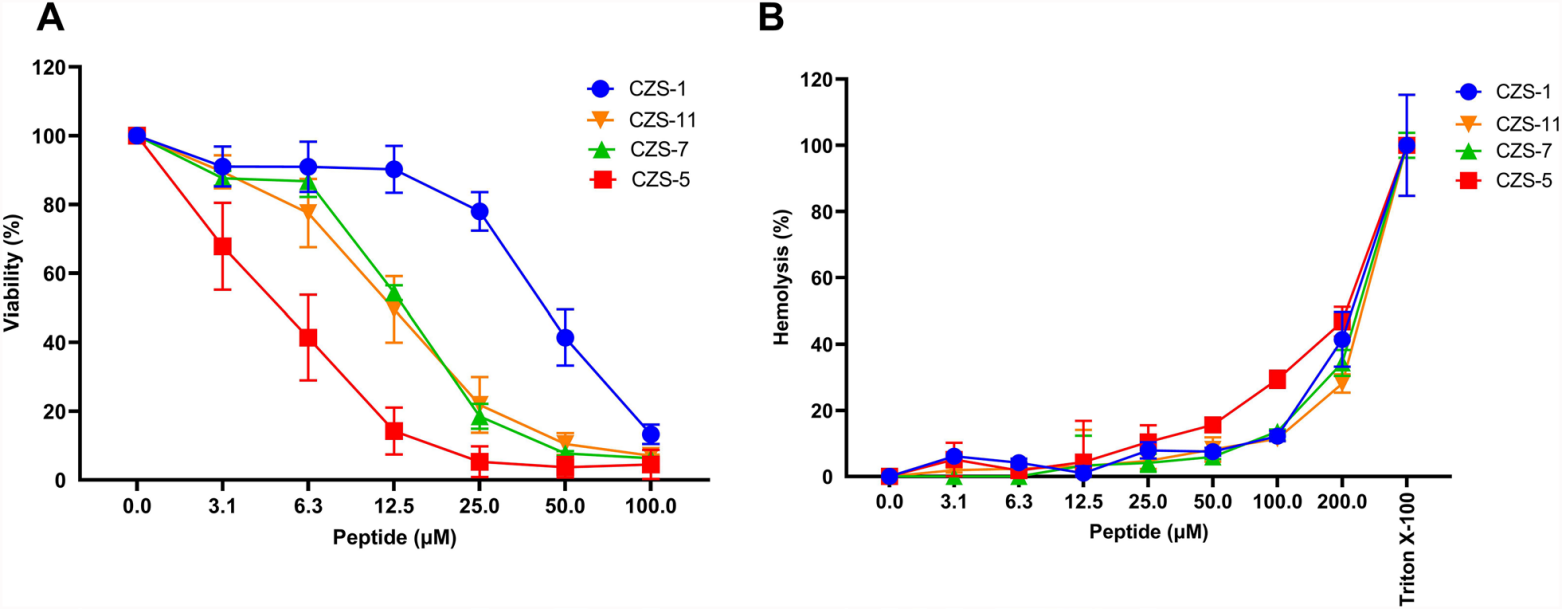
Effects of the tested cruzioseptins on *T. cruzi* X-1081 epimastigote viability and hemolysis of human erythrocytes. Epimastigotes of *T. cruzi* X-1081 were added in triplicate to 96-well microplates containing the indicated peptide concentrations and then incubated overnight at 28 °C. Resazurin viability assays were used considering control parasites without treatment as 100% viability. The experiment was repeated three times (*n* = 3). **B** Hemolysis assay conducted with human erythrocytes

Synthetic peptides were tested to assess their impact on *T. cruzi* epimastigote viability. Resazurin viability assays revealed differential effects among the tested cruzioseptins (Fig. 2A). CZS-5 exhibited the most potent anti-epimastigote effect, as indicated by its IC_50_ value (Table 2), followed by CZS-11, CZS-7, and CZS-1. The effect of CZS-5 on *T. cruzi* X-1081 epimastigotes was greater than that of the antichagasic drug benznidazole (IC_50_ = 13.3 ± 0.8 µM). The selectivity of these peptides was evaluated using hemolysis assays with human erythrocytes. Hemolytic effects are observed when the cruzioseptin concentration exceeds 50 μM (Fig. 2B), corresponding to a CC_50_ greater than 237.6 μM (Table 2). The selectivity index (SI) was calculated for each peptide as the ratio between the CC_50_ value (hemolysis) and the IC_50_ value (epimastigote viability) (Table 2). All peptides demonstrated selective effects against epimastigotes (SI > 1.0), with CZS-5 showing the highest selectivity. Consequently, CZS-5 was selected for further in-depth characterization.

**Table 2:** The activity of the peptides is expressed as half inhibitory concentrations for epimastigote viability (IC_50_) and for hemolysis (CC_50_)

Peptide	IC_50_ (µM)	CC_50_ (µM)	SI (CC_50_/IC_50_)
CZS-1	45.3 ± 4.9	386.4 ± 34.2	8.5
CZS-11	12.7 ± 2.9	567.5 ± 44.9	44.7
CZS-7	13.2 ± 0.8	470.7 ± 33.6	35.7
CZS-5^*^	4.7 ± 1.0	237.6 ± 15.2	50.3

The selectivity index (SI) was calculated as the ratio of the CC_50_ to the IC_50_

^*^CZS-5 was the most potent and selective peptide. Except for CZS-11 and CZS-7, all the peptides showed statistically significant differences in their IC_50_ and CC_50_ values (*P* ≤ 0.05, two-tailed parametric unpaired *t*-tests)

### CZS-5 kills different *T. cruzi* strains and compromises epimastigote membrane integrity

**Fig. 3:**
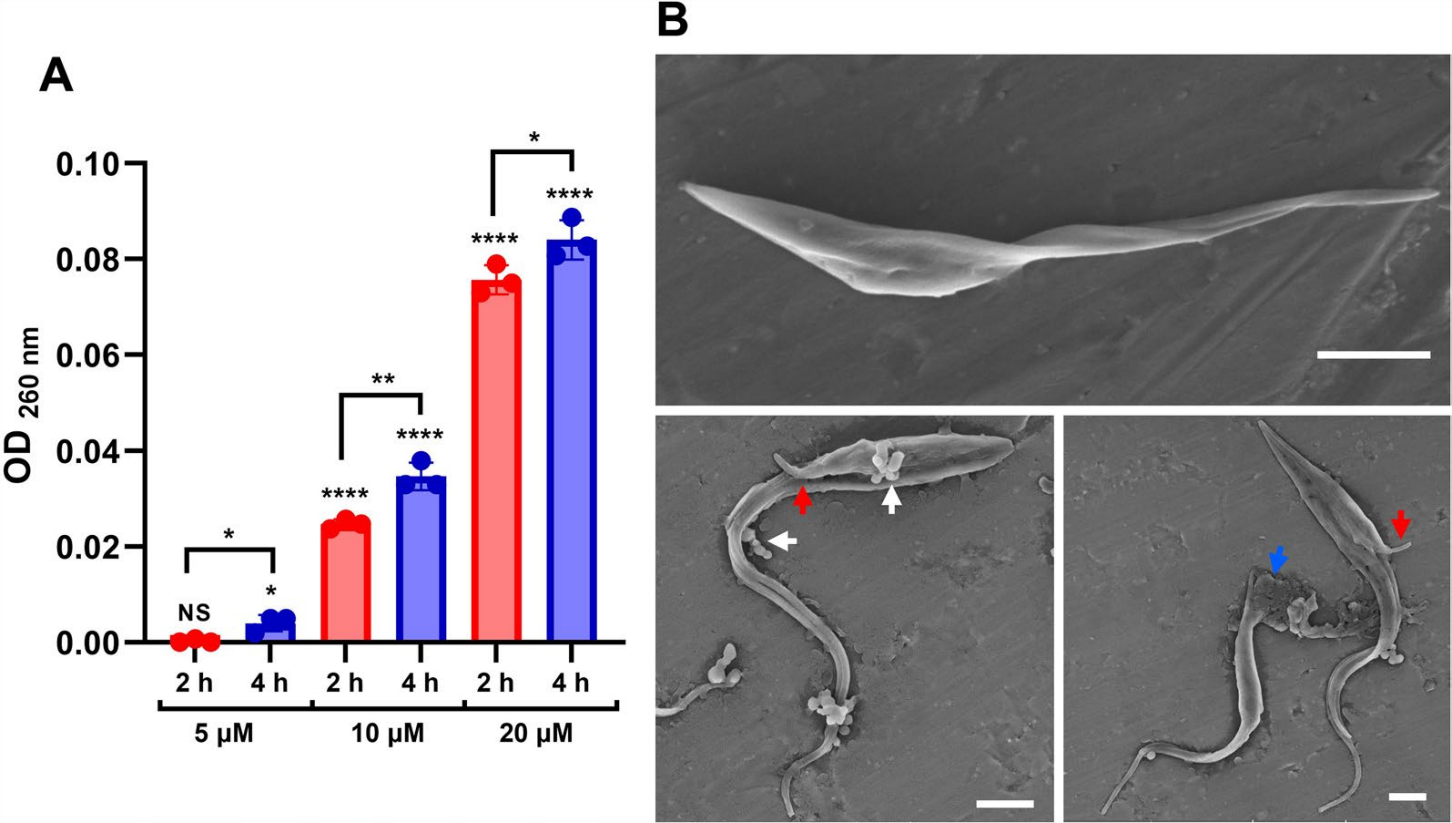
CZS-5 produces membrane alterations in *T. cruzi* epimastigotes. **A** Nucleic acid leakage assay of epimastigotes after treatment with different concentrations of CZS-5 (5 μM, 10 μM, and 20 μM) at two time points (2 h and 4 h). Comparisons of each treatment with the untreated control and of each concentration at the two tested times were conducted. Statistical significance was computed using two-tailed parametric unpaired *t*-tests, with the following results: NS = nonsignificant; *P ≤ 0.05; **P ≤ 0.01; ****P ≤ 0.0001. **B** SEM analysis of epimastigotes treated with CZS-5 5 μM for 4 h. Arrows indicate regions where membrane blebbing (white), flagellar detachment (red), or cell rupture (blue) suggest membrane damage. Scale bar = 2 μm

CZS-5 reduced the viability of epimastigotes from four different *T. cruzi* strains, representing both the TcI and TcII groups (Supplementary Fig. S1). The effect of CZS-5 was sequence dependent, as a scrambled version of CZS-5 (VQFVHSMGQNLAGNGDNVAKELKDVPIKAVKA) had no effect on parasite viability (Supplementary Fig. S1). To determine whether CZS-5 exerts its effect through membrane disruption, nucleic acid release from epimastigotes was monitored by measuring the absorbance at 260 nm following treatment for 2 and 4 h with various CZS-5 concentrations (Fig. 3A). The results indicated dose- and time-dependent release of nucleic acids, suggesting that CZS-5 compromises the parasite membrane. SEM analysis of epimastigotes treated with CZS-5 at 5 μM for 4 h further supported these findings, revealing membrane blebbing, cell rupture, and flagellar detachment from the cell body (Fig. 3B), all of which are consistent with membrane damage.

### CZS-5 treatment induces metabolomic alterations in epimastigotes

To gain further insight into the mode of action of CZS-5 and the parasite’s response to it, an untargeted metabolomics analysis was conducted. This analysis aimed to assess the changes in the metabolomic profile associated with CZS-5 exposure (5 µM) at two different time points (2 and 4 h). Untreated parasites or those treated with the scrambled CZS-5 peptide at 5 µM for 4 h were used as controls.

**Fig. 4:**
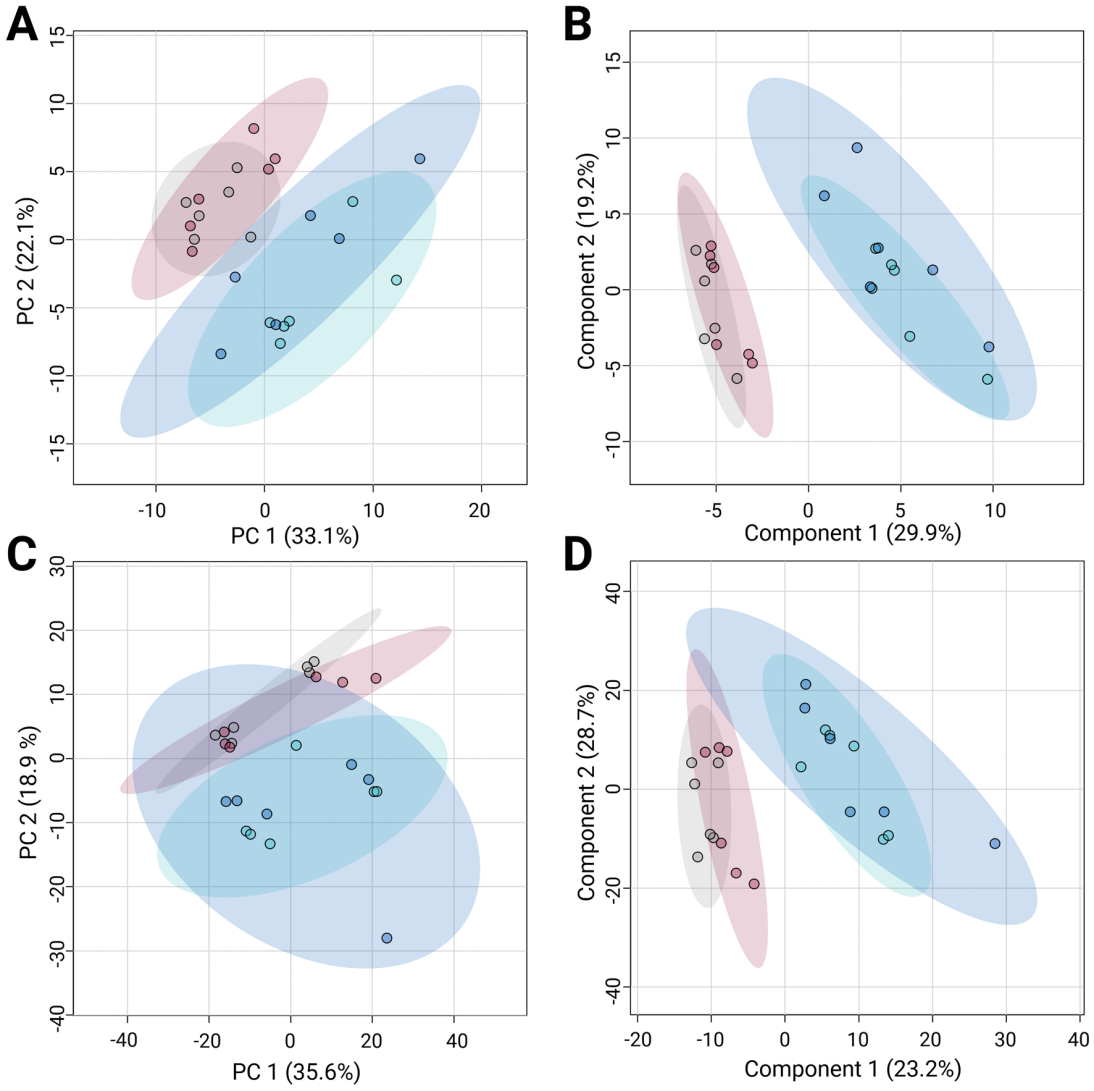
Multivariate models with unit variance (UV) scaling for metabolic analysis of treated parasites and the untreated control group. **A** and **C** PCA models. **B** and **D** PLS-DA models. **A** GC/MS, *R*^2^: 0.738. **B** GC/MS, *R*^2^: 0.932, *Q*^2^: 0.771, cv-ANOVA: 0.0005. **C** HILIC/MS, *R*^2^: 0.76. **D** HILIC/MS, *R*^2^: 0.99, *Q*^2^: 0.93, cv-ANOVA: 0.02. Gray, red, blue, and dark blue dots denote CTR, scrambled CZS-5, CZS-5 2 h, and CZS-5 4 h, respectively

A multiplatform strategy that combines gas and liquid chromatography with high-resolution mass spectrometry was employed to maximize metabolite detection exhibiting alterations. Examination of the clusters in the PCA models revealed the grouping of quality control samples across the GC/MS and HILIC/MS platforms (Supplementary Fig. S2A and B), suggesting analytical stability across all the platforms utilized. After verifying the performance of each analytical platform, both unsupervised PCA and supervised PLS-DA analyses were employed to identify trends, and the molecular characteristics significantly contributing to the separation of the untreated control (CTR), with those treated with the scrambled CZS-5 peptide, and with CZS-5 at 2 and 4 h (Fig. 4).

The PCA scoring plot shows adequate clustering of samples within each group across all implemented analytical platforms (Fig. 4A and C). In addition, similarities in metabolic characteristics were observed between the CTR and scrambled groups (represented by gray and red dots, respectively) and between the CZS-5-treated cultures at 2 and 4 h (represented by blue and dark blue dots, respectively). These trends remained consistent in the supervised PLS-DA models (Fig. 4B and D). Given the high similarity between the CTR and scrambled groups, OPLS-DA models were implemented to search for metabolic differences between these groups (Supplementary Fig. S3A and B, represented by gray and red dots, respectively). However, the generated models did not present acceptable statistical values (Q^2^: −0.242, 0.065; cross-validation variance [cv-ANOVA] > 0.05), suggesting that treatment with scrambled CZS-5 does not induce significant metabolic changes in the treated parasites under the tested conditions. This, in turn, supports the importance of the specific amino acid sequence in CZS-5 for its trypanocidal function.

Across both analytical platforms, the PLS-DA scoring plot demonstrated consistent clustering between the CTR (gray dots) and CZS-5 (blue and dark blue dots) groups (Fig. 4). Furthermore, adequate results were obtained for the metrics R^2^ and Q^2^, which assess the model’s goodness of fit and predictive power based on the data, respectively (*R*^2^: > 0.932; *Q*^2^: > 0.77). Cross-validation variance (cv-ANOVA) was used to confirm significant models across all platforms (cv-ANOVA < 0.05). This finding indicates that the multivariate models avoided overfitting [40]. Interestingly, the PLS-DA models revealed a clear separation between the CTR group and those treated with CZS-5 at 2 and 4 h, indicating distinct metabolic profiles for each of these groups (Fig. 4B and D).

**Fig. 5:**
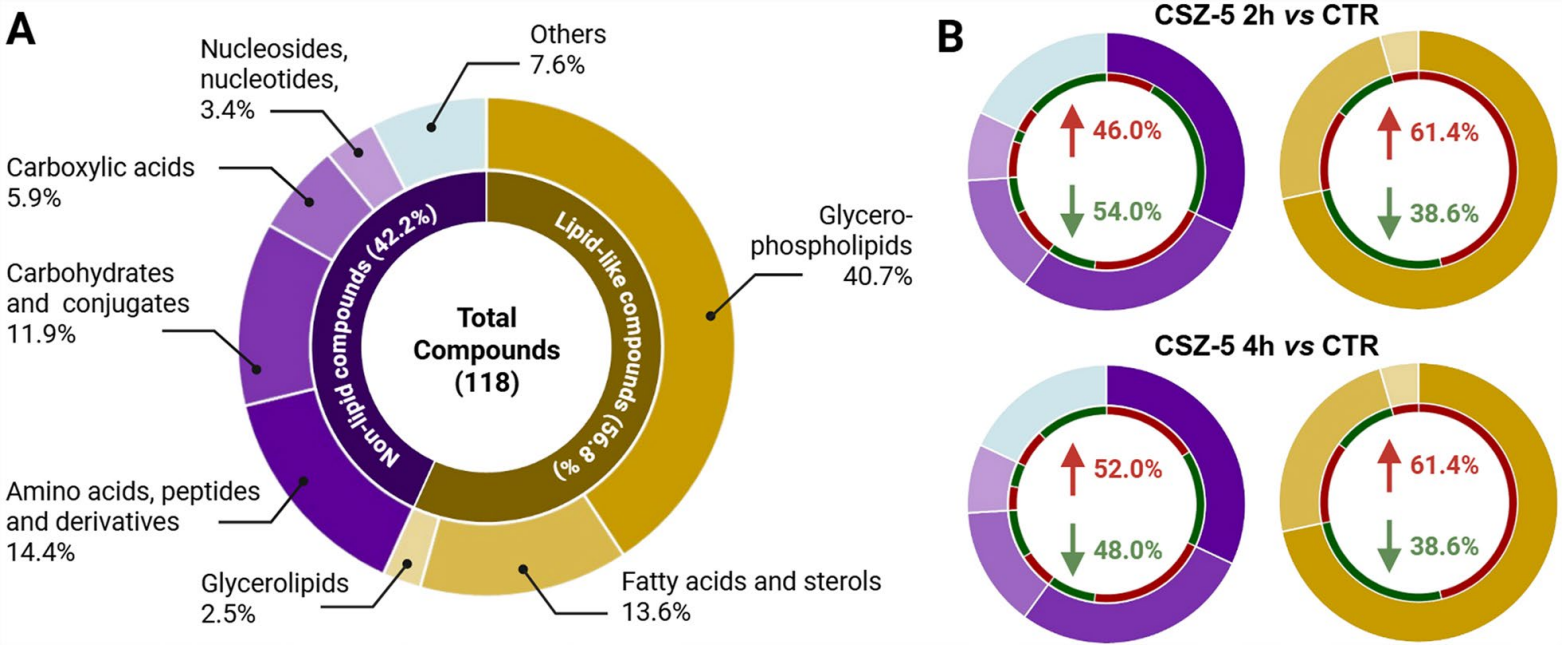
Altered metabolites among CTR, CZS-5 2 h, and CZS-5 4 h. **A** Total altered metabolites are classified according to their chemical classes. **B** Altered metabolites in the comparisons between CZS-5 2 h *versus* CTR and CZS-5 4 h *versus* CTR. The inner circle, colored red and green, represents metabolites whose levels are increased and decreased, respectively

A combination of multivariate analysis with a VIP threshold greater than 1, and univariate analysis with a significance level (*P*-value) adjusted for an FDR of less than 0.05 was used to identify specific distinguishing metabolites across the comparison groups. This approach led to the identification of 118 altered metabolites, covering all the platforms used in the study (Fig. 5; Supplementary Table S1). A total of 56.8% of the 108 changed metabolites were from lipid-related chemical families, with glycerophospholipids (40.7%), fatty acids (13.6%), and glycerolipids (2.5%) being the most affected. The remaining 42.2% was composed of nonlipidic chemical groups, including amino acids, peptides, and derivatives (14.4%), carbohydrates and conjugates (11.9%), carboxylic acids (5.9%), nucleosides and nucleotides (3.4%), and others (7.6%).

Supplementary Table S1 summarizes the altered metabolites identified in the treated parasites, providing details such as retention times, the coefficient of variation of the chromatographic signal in the QC group, the statistical parameters used for their selection, potential adducts, fold changes, and the type of confirmation, among other information. The analysis based on the chemical nature of the metabolites (lipid-like or nonlipid compounds) revealed similar trends in the changes observed between the comparisons of CZS-5 2 h *versus* CTR and CZS-5 4 h *versus* CTR. As shown in Fig. 5B, consistent trends were observed for lipid-type metabolites in both comparisons. In contrast, some shifts in trends were noted, particularly for metabolites such as amino acids, peptides, and their derivatives, with a greater proportion of them decreasing in the early hours.

**Fig. 6:**
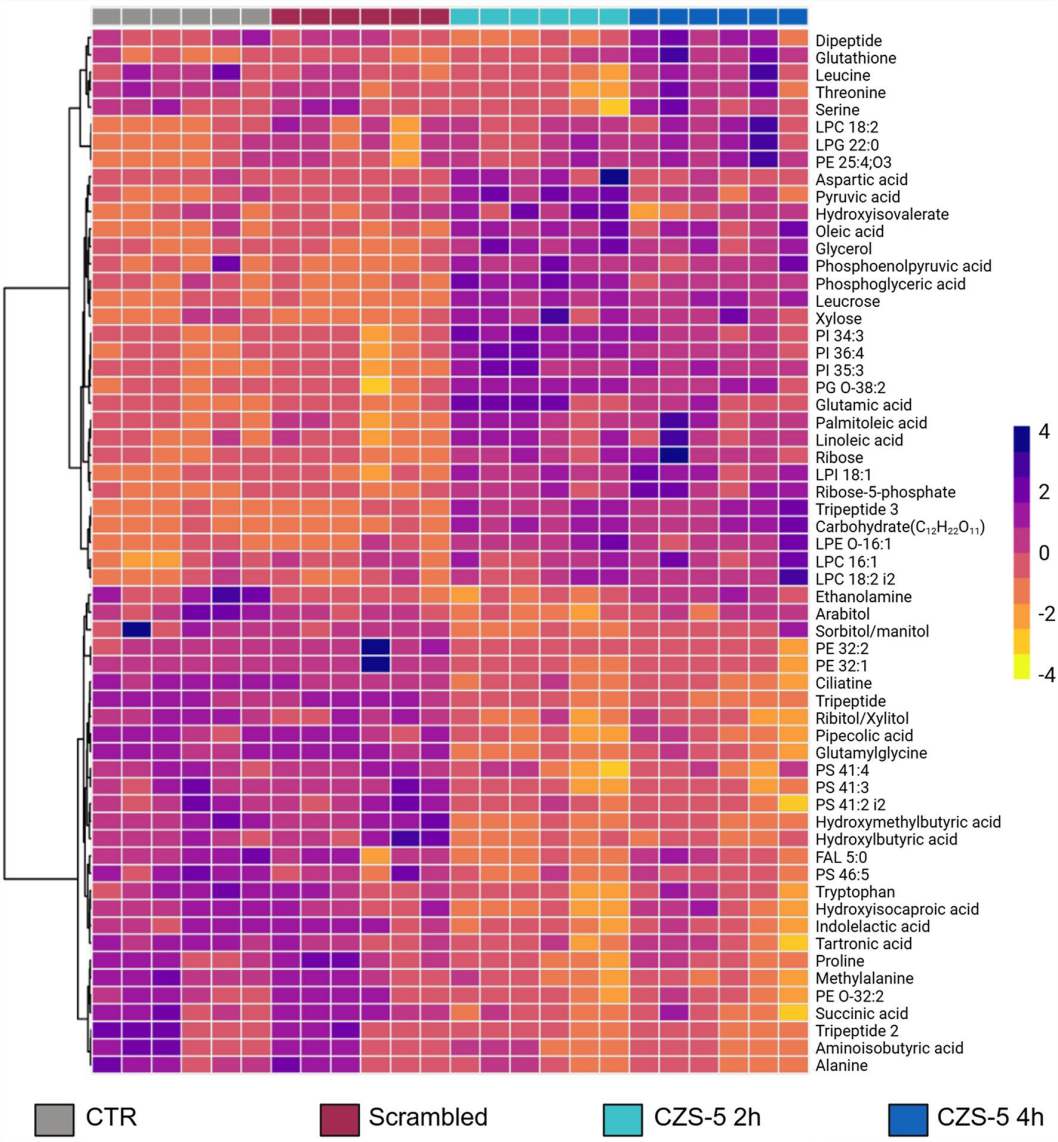
Heatmap of the top 60 altered metabolites showing statistically significant differences between *T. cruzi* epimastigotes treated with CZS-5, scrambled CZS-5, and CTR. The rows represent each altered metabolite identified, while the columns correspond to the analyzed samples, grouped by the following conditions: untreated parasites (gray), parasites treated with scrambled CZS-5 (red), and parasites treated with CZS-5 for 2 h (blue) and 4 h (dark blue). The level of variation is indicated on the right side by a color intensity scale representing relative abundance, where violet colors indicate an increase in metabolite levels, and yellow colors indicate a decrease

The comparison of individual metabolites using heatmaps allowed the visualization of metabolite patterns that changed between the experimental groups (Fig. 6). Yellow indicates decreased metabolite levels in the treated parasites, whereas violet indicates increased metabolites (Fig. 6). The presented heatmap reveals a notable similarity in the altered metabolite levels between the CTR group and those treated with scrambled CZS-5 at 5 μM for 4 h, as well as between parasites treated with CZS-5 for 2 and 4 h, which supports the findings observed in the PLS-DA plots (Fig. 4B and D). The heatmap highlights the formation of two clades, with the upper clade displaying significantly higher levels of metabolites in parasites treated with CSZ-5. This clade is enriched primarily with lipids, including lysophospholipids (LPLs), phosphatidylinositols (PIs), and fatty acids such as oleic acid and hydroxy isovaleric acid. In addition, amino acids such as serine, threonine, leucine, and aspartic acid, along with peptides such as glutathione, were also found to be elevated. Furthermore, key intermediates in glycolysis and the biosynthesis of energy compounds, such as phosphoenolpyruvate, 3-phosphoglyceric acid, and pyruvate, were observed, along with intermediates from the pentose phosphate pathway, including ribose and ribose-5-phosphate.

In contrast, the lower clade presented decreased metabolite levels in CZS-5-treated parasites. This reduction is primarily observed in chemical families such as phosphatidylethanolamines and phosphatidylserines, as well as certain amino acids (*i.e.,* tryptophan, alanine, and proline). The described results suggest the involvement of two metabolic processes in CSZ-5-treated parasites: alterations in energy production and in membrane composition. These findings highlight key changes in metabolic regulation, which may be related to the adaptation of the parasites to the conditions induced by the treatment.

### Molecular dynamics simulations

CG–MD simulations were performed to test the membrane-pore formation potential of cruzioseptin peptides in a *T. cruzi* epimastigote-like lipid bilayer. MD simulations started with a peptide/lipid ratio close to 0.03. Similar peptide/lipid ratios for other antimicrobial peptides have been shown to induce leakage in liposomes [30–32]. Here, all the minimized peptides were inserted into the membrane and oriented vertically to address whether the peptide has the propensity to form oligomers that eventually lead to a membrane pore.

**Fig. 7:**
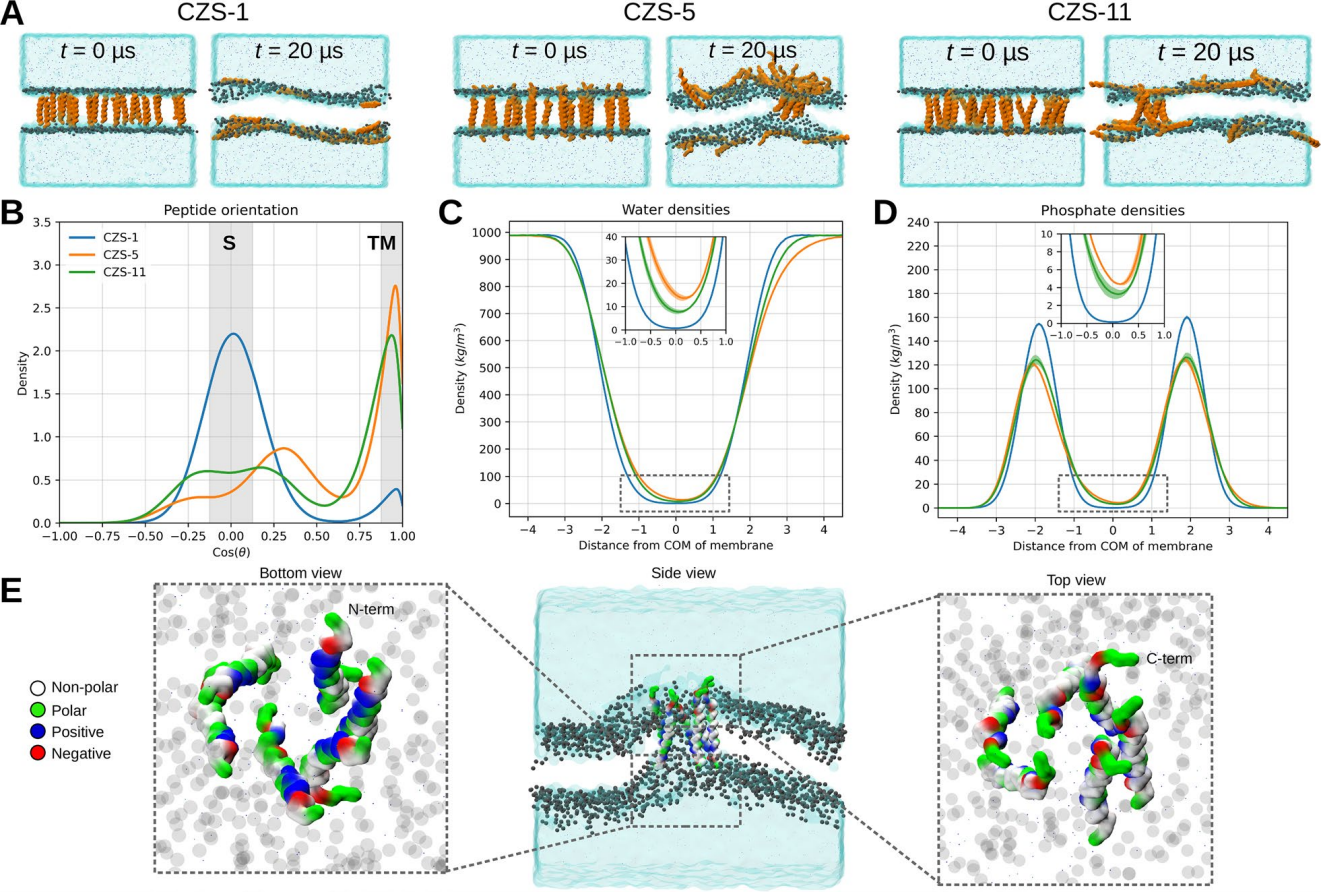
CG‒MD simulations of membrane-embedded cruzioseptins. **A** Representative snapshots from one replicate showing the initial and final confirmation of each simulated peptide. The peptides are shown as orange surfaces, while the lipid phosphate groups are shown as gray spheres; lipid acyl tails are omitted for clarity. Water is represented as a transparent light blue surface, and NaCl ions are depicted as dots. **B** Peptide orientation was determined by measuring the angle of each peptide relative to the normal vector. The superficial (horizontal, *θ* = 90°) and transmembrane (vertical, *θ* = 0°) orientations are highlighted with gray shading labeled S and TM, respectively. **C** and **D** Density plots for the water and phosphate distances from the center of mass (COM) of the membrane, respectively. The solid lines represent the average, whereas the light-colored areas indicate the standard deviation across three independent replicates. Data from all peptide copies and replicas were concatenated to construct each histogram. **E** Schematic representation of the transmembrane hexamer formed by CZS-5. The peptides are shown as colored surfaces, where the negatively charged, positively charged, polar, and nonpolar amino acids are shown in red, blue, green, and white, respectively. The phosphate beads and the water are represented as in (**A**) the bottom and top views of the hexamer are shown for visual reference of the architecture of the pore

Three cruzioseptin peptides were simulated: a peptide with high activity against epimastigotes (CZS-5, IC_50_ = 4.7 μM ± 1.0), a peptide with medium activity (CZS-11, IC_50_ = 12.7 μM ± 2.9), and a peptide with low activity (CZS-1, IC_50_ = 43.3 μM ± 4.9) toward the parasite. CG-MD simulations revealed that CZS-1 leaves the interior of the lipid membrane and positions itself on the membrane surface (S state), whereas the CZS-5 and CZS-11 peptides resulted in some oligomers whose transmembrane (TM) position was preserved (Fig. 7A and B).

To quantify the position of the peptides in the membrane, we first calculated their tilt angle so we were able to distinguish between superficial (S) and TM orientations. The angles obtained (Fig. 7A and B) confirmed that CZS-1 was positioned on the surface of the membrane. This peptide shows a completely horizontal orientation or an angle of 90° (S state in Fig. 7B and Supplementary Fig. S4A), which is consistent with CZS-1 interacting with the phospholipid (PL) headgroups located at the edges of the membrane. Conversely, CZS-5 and CZS-11 were able to form stable transmembrane oligomers (Supplementary Fig. S4B). This preference for a TM state can also be observed when inspecting their angles, where CZS-5 was the peptide most prone to orient itself at an angle of ~0° < θ <  ~20°, which leads to a TM state (Fig. 7B and Supplementary Fig. S4).

The formation of oligomers of antimicrobial peptides in the TM orientation is expected to disrupt membrane permeability, which at the molecular level translates into solvent flow across the membrane. As observed in our simulations by measuring the water density for all the systems and using the center of mass of the membrane as a reference point, the CZS-5 peptide allowed the highest amount of water flux across the membrane (Fig. 7C), followed by CZS-11 and finally the CZS-1 peptide with almost no water flow. By measuring the lipid phosphate bead density, we observed effects similar to those of water, with CZS-5 being the peptide that induced the most lipid mixing, followed by CZS-11, and minimal lipid mixing.

Our simulations suggest that CZS-5 tends to form larger TM oligomers than CZS-11 since CZS-5 forms heptamers, hexamers, and trimers in its three replicates. Instead, CZS-11 formed dimers, trimers, tetramers, and pentamers in its three replicates (Supplementary Fig. S4B). This observed behavior is supported by the fact that by computing the RDFs of the peptides, the results demonstrate that CZS-5 has a greater propensity to form oligomers than CZS-11 (Supplementary Fig. S4C). Interestingly, one of the CZS-5 replicas led to a TM hexamer (Fig. 7E), which presented most of its polar/charged residues (hydrophilic face of the peptide: Asp4, Lys7, His8, Lys11, Lys15, Ans22, and Ans26) facing toward the pore, which is in line with water flowing across the membrane. As a result, most of the nonpolar residues of the hexamer (hydrophobic face of the peptide: Phe2, Val5, Ile6, Val9, Val13, Ala17, Ala20, and Val21) face the lipid core of the membrane. From this final conformation, it was observed that the bottom and top sides of the pore presented different sizes, being ~2.6 nm at the bottom side (where the N-terminals are) and ~2.3 nm at the top side (where the C-terminals are) (Fig. 7E). The smaller size at the top side of the hexamer could be explained by the fact that the C-terminal (the last 6 amino acids, from Lys27 to Ser32) of CZS-5 is predicted to be a coil, which would reduce the stability of the peptide‒peptide interaction at this side of the oligomer. However, because of the limitations of the resolution and settings (*i.e.,* peptide/lipid ratio, lipid composition, and fixed secondary structure) of our simulations, these sizes require rigorous and further validation.

## Discussion

**Fig. 8:**
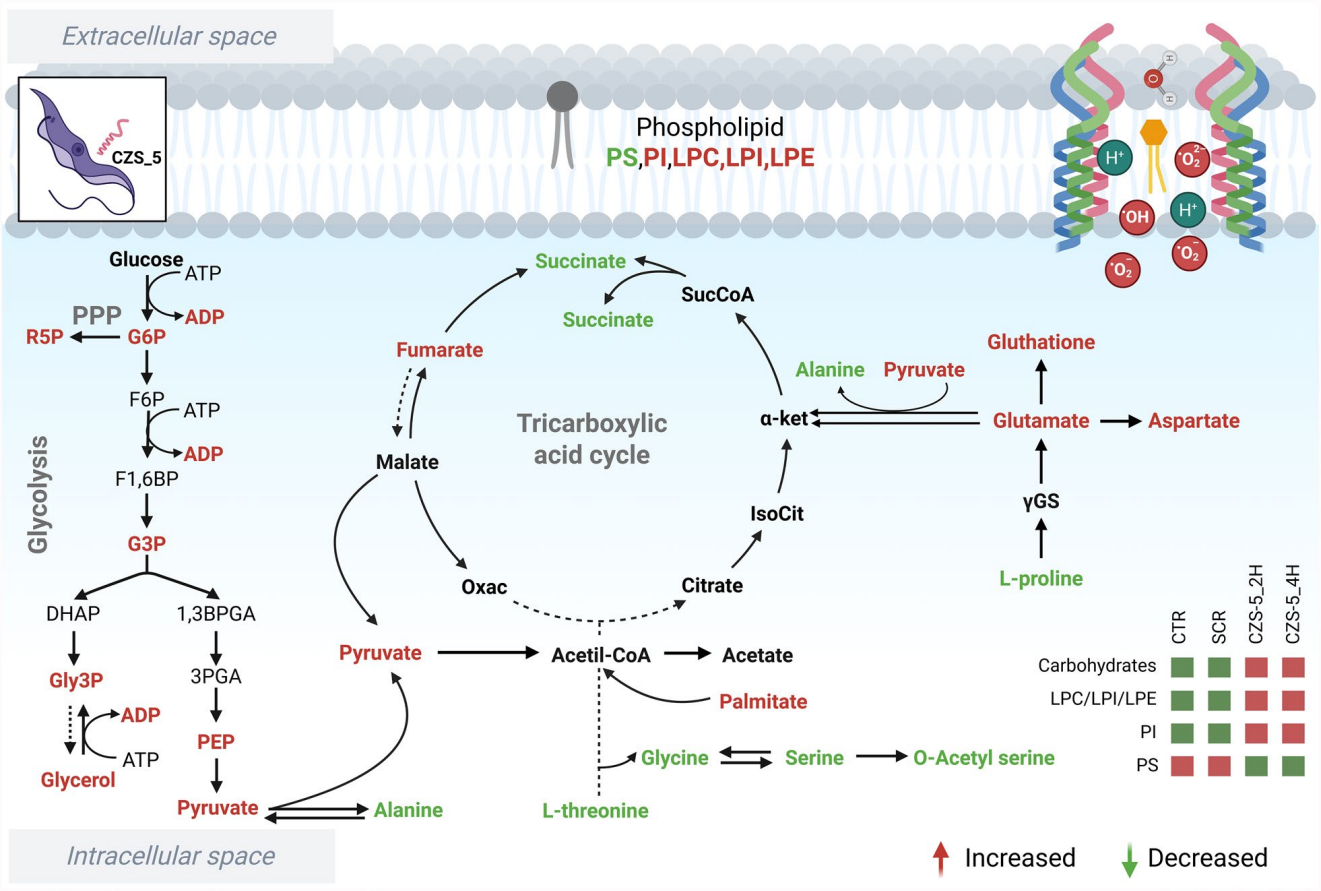
Altered metabolism in *T. cruzi* epimastigotes treated with CZS-5. The figure highlights the main metabolic alterations observed in the comparisons. Metabolites showing significant changes are highlighted in red or green, indicating increased or decreased levels, respectively. Created in BioRender

There is an urgent need for novel treatments for Chagas disease, and antimicrobial peptides from diverse sources have been suggested as promising therapeutic alternatives [6, 9, 14, 41–43]. This study demonstrated the selective anti-*T. cruzi* effect of cruzioseptins, a family of 17 AMPs secreted by the skin of the neotropical frog *Cruziohyla calcarifer* [10]. Herein cruzioseptins were divided into two groups, named groups 1 and 2, on the basis of the presence of a specific amino acid insertion and several substitutions. Previously, anti-parasitic effects for cruzioseptins from group 1 (CZS-1 to 4 and CZS-16) were demonstrated for *Leishmania* spp. and *T. cruzi* [12, 13]. In this work, CZS-1, which belongs to group 1, and three cruzioseptins from group 2 (CZS-5, CZS-7, and CZS-11) were tested against epimastigotes of *T. cruzi*. CZS-5 (IC_50_ = 4.7 μM ± 1.0) proved to be more potent than the antichagasic drug benznidazole (IC_50_ = 13.3 ± 0.8 µM) against *T. cruzi* X-1081 epimastigotes [21], a strain belonging to the TcI group, which is among the most predominant in human infections [44]. In addition, among the evaluated peptides CZS-5 was the most selective, with an SI > 50, which meets the World Health Organization (WHO)/TDR criterion for being considered a potential drug candidate [45]. Mechanistic studies provide strong evidence that CZS-5 causes membrane damage, as it induces DNA leakage and surface disruption in epimastigotes. Furthermore, molecular dynamics simulations of CZS-5 embedded in model epimastigote lipid bilayers suggest that the peptide may form stable aggregates with pore-like structures, as previously suggested by docking studies with other cruzioseptins embedded in model eukaryotic membranes [13]. Finally, nontargeted metabolomic analysis of CZS-5-treated epimastigotes revealed several altered metabolites suggesting that, in addition to membrane disruption, peptide-induced parasite killing involves a complex metabolic response (Fig. 8).

Although this study was limited to the epimastigote stage, this model was selected owing to its experimental advantages and the exploratory nature of the research. *T. cruzi* has a complex life cycle comprising infective and non-infective stages for the human host [46]. These stages exhibit profound morphological, metabolic, gene expression, and membrane composition differences, which may influence their susceptibility to pharmacological compounds. Therefore, it is highly recommended to also perform their evaluation in trypomastigotes and intracellular amastigotes [47], life stages of greater clinical relevance. In the case of AMPs, this is particularly important due to their relatively large molecular size, which can limit their ability to act on intracellular stages. Despite this, numerous AMPs have been reported to selectively affect intracellular stages in several parasite species [9], including cruzioseptins against *T. cruzi* and *Leishmania* spp. [12, 13]. Furthermore, a major advantage of AMPs is their amenability to chemical modification to enhance properties such as host cell entry [9]. In this study on the mechanism of action of CZS-5, we used epimastigotes as a model owing to their ease of culture and the ability to obtain large and reproducible amounts of biomass [25, 48, 49]. This is critical in untargeted metabolomics, where broad coverage and sensitivity are required to confidently detect most altered metabolites induced by a given treatment. The results obtained in epimastigotes are valuable as they can inform future studies on other life stages, focused on specific metabolite groups and requiring fewer parasites. Moreover, the mechanisms described here can guide the rational design of AMPs with optimized anti-*T. cruzi* properties, such as improved selectivity and host cell penetrability.

AMPs are recognized for their broad-spectrum antimicrobial activity, which is attributed to diverse mechanisms of action that are difficult to unravel [50]. Metabolomics detects changes in individual metabolites or pathways induced in a cell by treatment with a drug or drug candidate, helping to elucidate its mode of action [18]. This approach has been used to study the mode of action of diverse anti-protozoal drugs, such as the trypanocidal agent benznidazole [49, 51]. AMPs generally target the parasite membrane, promoting membrane permeabilization and leading to cell death [6, 9, 14]. In contrast to healthy human cells, pathogen membranes are characterized by the presence of anionic glycerophospholipids on the outer leaflet of the plasma membrane [9]. This selective electrostatic attraction of cationic AMPs to the pathogen plasma membrane [52] is consistent with the observed effects of CZS-5 on epimastigote glycerophospholipids (PIs, PSs, and PGs) and LPLs (LPGs and LPIs) with anionic polar headgroups. Once bound on the membrane surface, the peptide may have at least two distinct nonexclusive destinations [14]: (1) disrupting the parasite membrane, and (2) gaining access to the parasite cytoplasm and interfering with intracellular processes.

Nucleic acid leakage assays revealed a time- and dose-dependent damaging effect of CZS-5 on the epimastigote membrane. SEM observations of CZS-5-treated epimastigotes revealed membrane blebbing, cell rupture, and flagellar detachment, all of which are indicative of peptide-induced membrane disruption [53]. This finding aligns with the primary mode of action observed in other peptides with activity against different life stages of *T. cruzi*, including trialysin, Def1.3, cecropin A, Def-α-1, melittin, BatxC, Ctn, and other cruzioseptins [13, 16]. AMPs have been shown to induce parasite death through mechanisms such as targeting intracellular calcium stores, disrupting mitochondrial function, and interfering with gene expression [6, 14–16]. While these mechanisms were not explored herein for CZS-5, they cannot be ruled out.

There are two main modes of membrane damage caused by cationic, linear, and α-helical antimicrobial peptides such as cruzioseptins [9, 50, 54]. First, the carpet-like model claims that AMPs extensively cover the membrane surface, leading to cell lysis by detergent-like membrane disruption. Second, AMPs can insert into the acyl chain region of the lipid bilayer to form aggregates and constitute a pore whereby ions, nutrients, and large cytoplasmic components escape. The precise mode of action of a peptide depends on its physicochemical and structural properties, the features of the target membrane, and the environmental conditions [54, 55]. The detailed molecular mechanisms of AMP-mediated membrane damage have been described for only a few peptides [17]. Molecular docking with a modeled eukaryotic membrane suggests that cruzioseptins may form pores in these membranes [13]. To further investigate this mechanism, a model epimastigote lipid bilayer was constructed on the basis of its lipid composition [34] and then used to perform coarse-grained molecular dynamic simulations of the different degrees of activity of the cruzioseptins in this parasite stage (CZS-5 > CZS-11 > CZS-1). Most of the CZS-1 peptides, the least active tested cruzioseptin, were expelled from their initial transmembrane conformation to one of the membrane leaflets. In contrast, both CZS-5 and CZS-11 remain inserted into the lipid bilayer and form stable pore-like aggregates.

Two models of pore formation by AMPs into the pathogen’s membrane have been proposed [50, 54]. In the barrel–stave model, amphipathic peptide helices inserted into the lipid bilayer form a bundle with its hydrophobic faces oriented toward the acyl chains of the glycerophospholipids and the polar amino acid side chains forming the pore channel. The toroidal pore structure is similar to that proposed by the barrel–stave model, but with the polar heads of the glycerophospholipids also being part of the pore wall. MD simulations performed with CZS-5 and CZS-11 revealed the movement of phosphate headgroups from the glycerophospholipids between both membrane leaflets, which is consistent with the toroidal model. MD simulations suggest that pores produced by CZS-5 and CZS-11 may vary in size and composition, as has been reported for other pore-forming AMPs [17, 54, 56, 57]. Interestingly, the observed flow of water and polar headgroups through the membrane pores formed by CZS-5 and CZS-11 was proportional to the anti-*T. cruzi* activity of these peptides. These findings suggest that the pore structure could influence the observed peptide antiparasitic effects.

Pore formation may be only one of the first steps in peptide-induced cell death [58]. One possible downstream event of CZS-5-mediated parasite membrane disturbance is the generation of reactive oxygen species (ROS) facilitated by the transfer of water and glycerophospholipids across the lipid bilayer. Oxidative stress in CZS-5-treated epimastigotes was evidenced by the alteration of several metabolites, including the increased amount of glutathione, which is key for the parasite ROS detoxification machinery [59]. This finding is further supported by the elevated levels of LPLs, especially after 2 h of treatment with CZS-5. LPLs are formed from PLs either via cleavage by phospholipases or through the actions of ROS that can be induced in pro-oxidant environments [25]. In cases of oxidative stress-induced cleavage, ROS have been shown to generate LPL through the hydrolysis (acidic or alkaline) of esterified fatty acyl chains [60, 61]. Increased production of ROS has also been documented in *T. cruzi* epimastigotes treated with BatxC, a cathelicidin-related AMP [62]. In addition, metabolic alterations, similar to those observed in CZS-5-treated epimastigotes, were observed in parasites subjected to glucose oxidase, an agent that induces oxidative stress through the continuous production of H_2_O_2_ [48]. Metabolic adaptations to this oxidative stress include upregulation of the pentose phosphate pathway, as indicated by elevated levels of intermediates such as pentoses and glucuronates, which are essential for producing NADPH to maintain redox homeostasis. Concurrently, a reduction in succinate—a key metabolite generated in glycosomes during carbohydrate metabolism—was observed, along with a moderate decrease in amino acids such as valine, glutamic acid, aspartic acid, and glycine, suggesting a decline in energy production through amino acid metabolism [48].

## Conclusions

Herein, an untargeted metabolomics strategy combined with molecular dynamics simulations using the epimastigote stage of *T. cruzi* as a model were used to provide insights into the mechanism of action of CZS-5, a potent member of the cruzioseptin family of AMPs. The peptide’s ability to disrupt membranes, alter diverse metabolites, and trigger oxidative stress suggests multiple mechanisms of action. For moving forward with the development of cruzioseptins as viable therapeutic candidates, the validation of the presented findings in clinically relevant forms of the parasite is crucial. The identified altered metabolites and the potential mechanisms of action identified for CZS-5 in epimastigotes lay the groundwork for further studies using other parasite stages. In addition, this information might be important for the future design of AMPs with improved properties.

## Supplementary Information

Supplementary Material 1: Fig S1. Effect of CZS-5 on epimastigotes of different *T. cruzi* strains. Parasite viability was determined using resazurin viability assays. Strains X-1081, Mg, and Ds were genotyped as TcI, whereas the Y strain belongs to the TcII group [21]. The IC_50_ values for each strain are indicated in parenthesis.

Supplementary Material 2: Fig S2. Multivariate PCA for untargeted metabolomic platforms. A GC/MS, R^2^: 0.806. B HILIC/MS, R^2^: 0.738. Gray and orange dots represent the samples and QCs, respectively.

Supplementary Material 3: Fig S3. Multivariate OPLS-DA for the comparison between the CTR and SCR groups. A GC/MS, R²: 0.76, Q²: 0.242, cv-ANOVA: 0.7. B HILIC/MS (-), R²: 0.688, Q²: -0.065, cv-ANOVA: 1. Gray and red dots represent the CTR and scrambled CZS-5, respectively.

Supplementary Material 4: Fig S4. Peptide orientations and oligomers after CG-MD simulations. A Angle distributions per simulated peptide and per replica. B Final conformation of the peptides that kept the TM orientation after CG-MD simulations. C Radial distribution, g(r), functions of the simulated peptides. The backbone bead used for g(r) calculations is shown as a gray bead located at the center of the corresponding peptide, thus 25 beads (1 bead per peptide) were used per calculation. For B and C, the peptides are shown as surfaces where the positive, negative, polar and non-polar amino acids are colored in blue, red, green and white, respectively.

Supplementary Material 5: Table S1. Altered metabolites between CTR and those treated with the peptide CZS-5 at 2 and 4 h.

## Data Availability

Data is provided within the manuscript or supplementary information. Additional information will be made available upon request.

## Abbreviations

AMP: Antimicrobial peptide
CC_50_: Half maximal cytotoxic concentration
CG-MD: Coarse-grained molecular dynamics
CTR: Untreated control
EDTA: Ethylenediaminetetraacetic acid
FBS: Fetal bovine serum
FDR: False discovery rate
GC-QTOF-MS: Gas chromatography/quadrupole time-of-flight mass spectrometry
GRAVY: Grand average of hydropathicity
HILIC-LC-QTOF-MS: Hydrophilic interaction liquid chromatography coupled to quadrupole time-of-flight mass spectrometry
IC_50_: Half maximal inhibitory concentration
LIT: Liver infusion tryptose
LPC: Lysophosphatidylcholine
LPE: Lysophosphatidylethanolamine
LPI: Lysophosphatidylinositol
LPG: Lysophosphatidylglycerol
LPL: Lysophospholipid
OPLS-DA: Orthogonal partial least squares discriminant analysis
PCA: Principal component analysis
PBS: Phosphate-buffered saline
PG: Phosphatidylglycerol
PI: Phosphatidylinositol
PL: Phospholipid
PLS-DA: Partial least squares discriminant analysis
PS: Phosphatidylserine
PPP: Pentose phosphate pathway
QC: Quality control
RP-HPLC: Reverse-phase high-performance liquid chromatography
ROS: Reactive oxygen species
SEM: Scanning electron microscopy
SI: Selectivity index
TM: Transmembrane
UV: Unit Variance
VIP: Variance important in projection
RDF: Radial Distribution Function
