# Supplementary figures and images for: Exploring the mechanisms of action of the antimicrobial peptide CZS-5 against Trypanosoma cruzi epimastigotes: insights from metabolomics and molecular dynamics
Source: Parasit Vectors. 2025 Jun 5;18:208. doi: 10.1186/s13071-025-06861-5 (PMC12139317; doi:10.1186/s13071-025-06861-5)

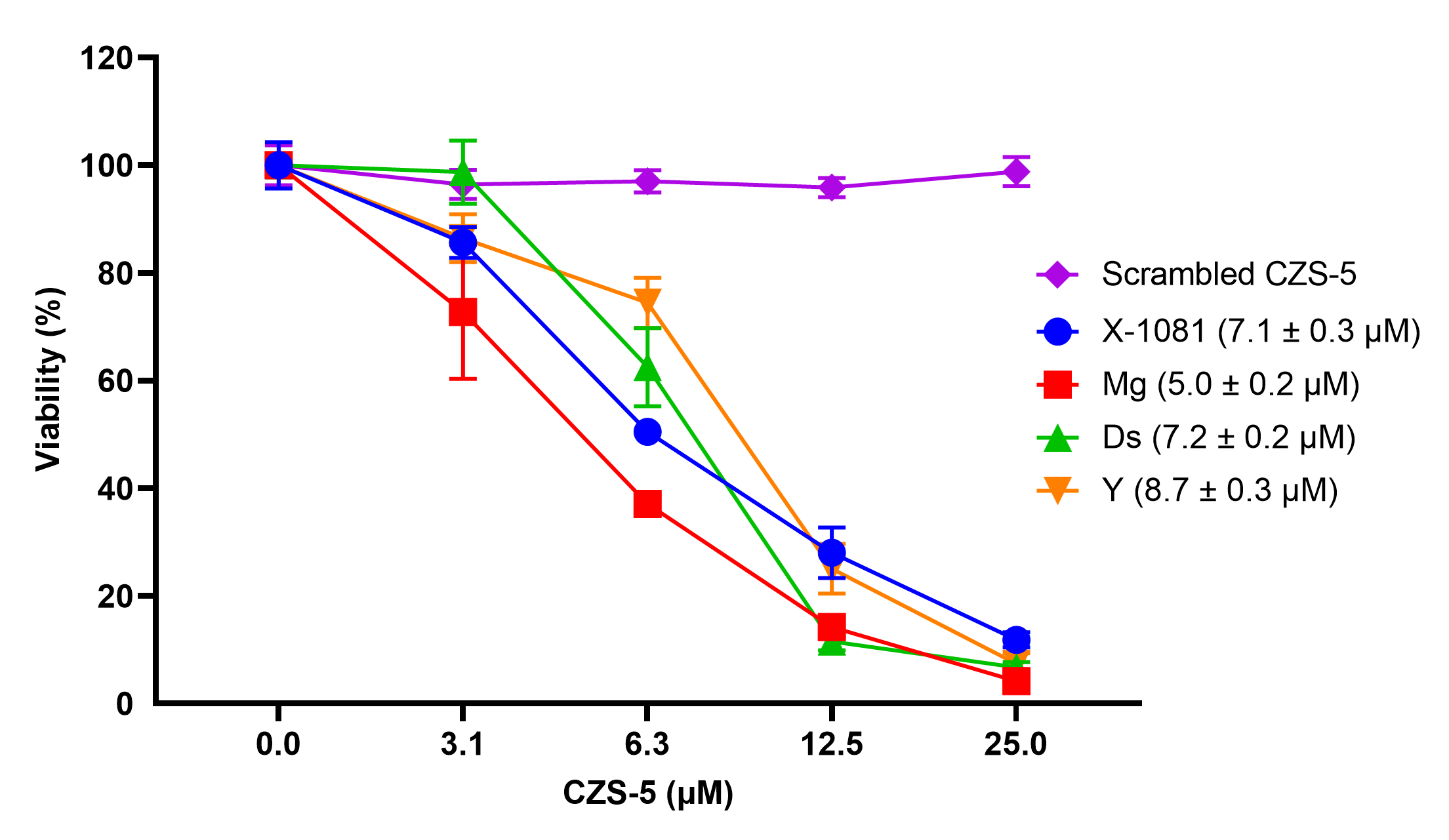

Supplement: Supplementary file 1 — Supplementary Material 1: Fig S1. Effect of CZS-5 on epimastigotes of different T. cruzi strains. Parasite viability was determined using resazurin viability assays. Strains X-1081, Mg, and Ds were genotyped as TcI, whereas the Y strain belongs to the TcII group [21]. The IC50 values for each strain are indicated in parenthesis. [file 13071_2025_6861_MOESM1_ESM.tif]

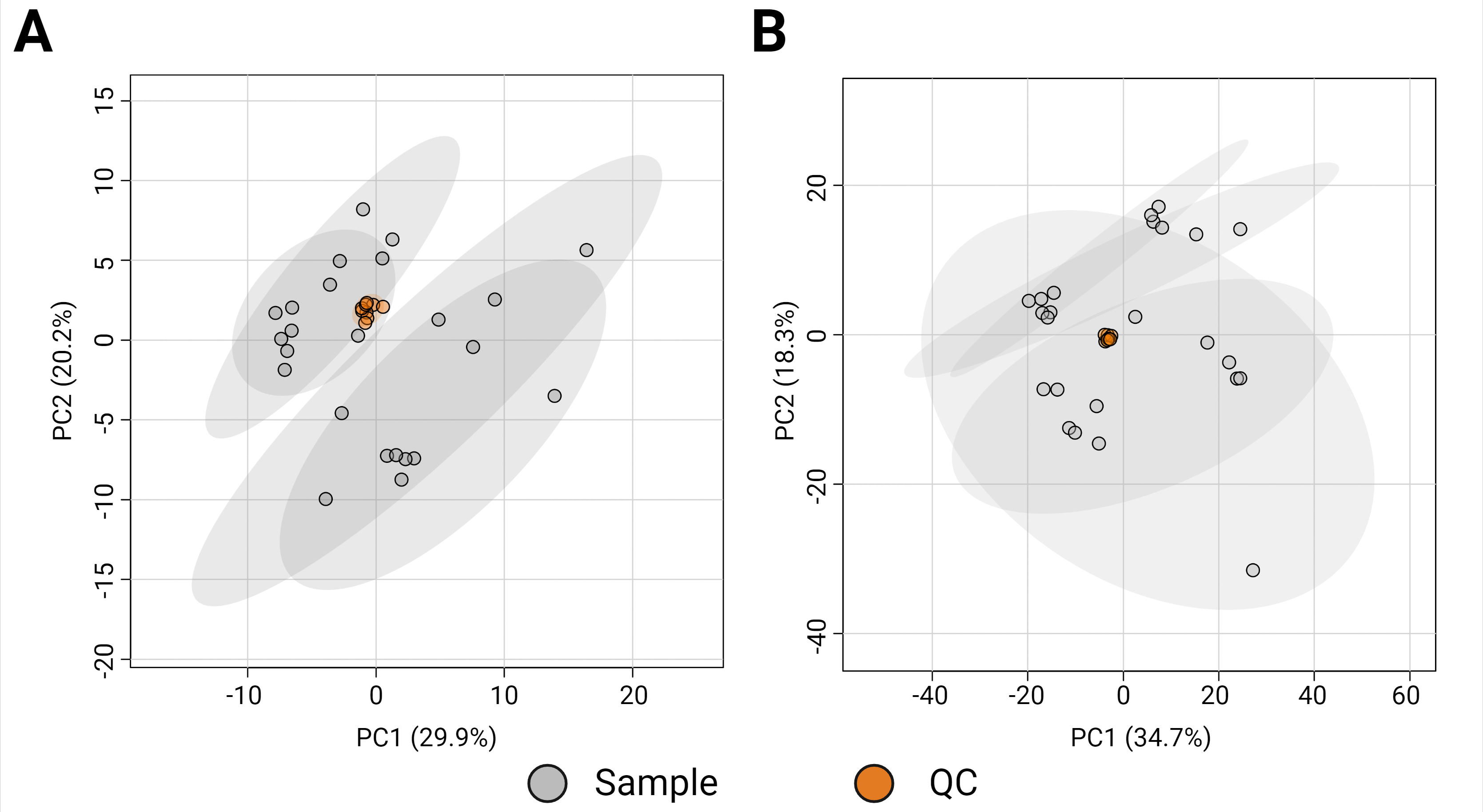

Supplement: Supplementary file 2 — Supplementary Material 2: Fig S2. Multivariate PCA for untargeted metabolomic platforms. A GC/MS, R2: 0.806. B HILIC/MS, R2: 0.738. Gray and orange dots represent the samples and QCs, respectively. [file 13071_2025_6861_MOESM2_ESM.tif]

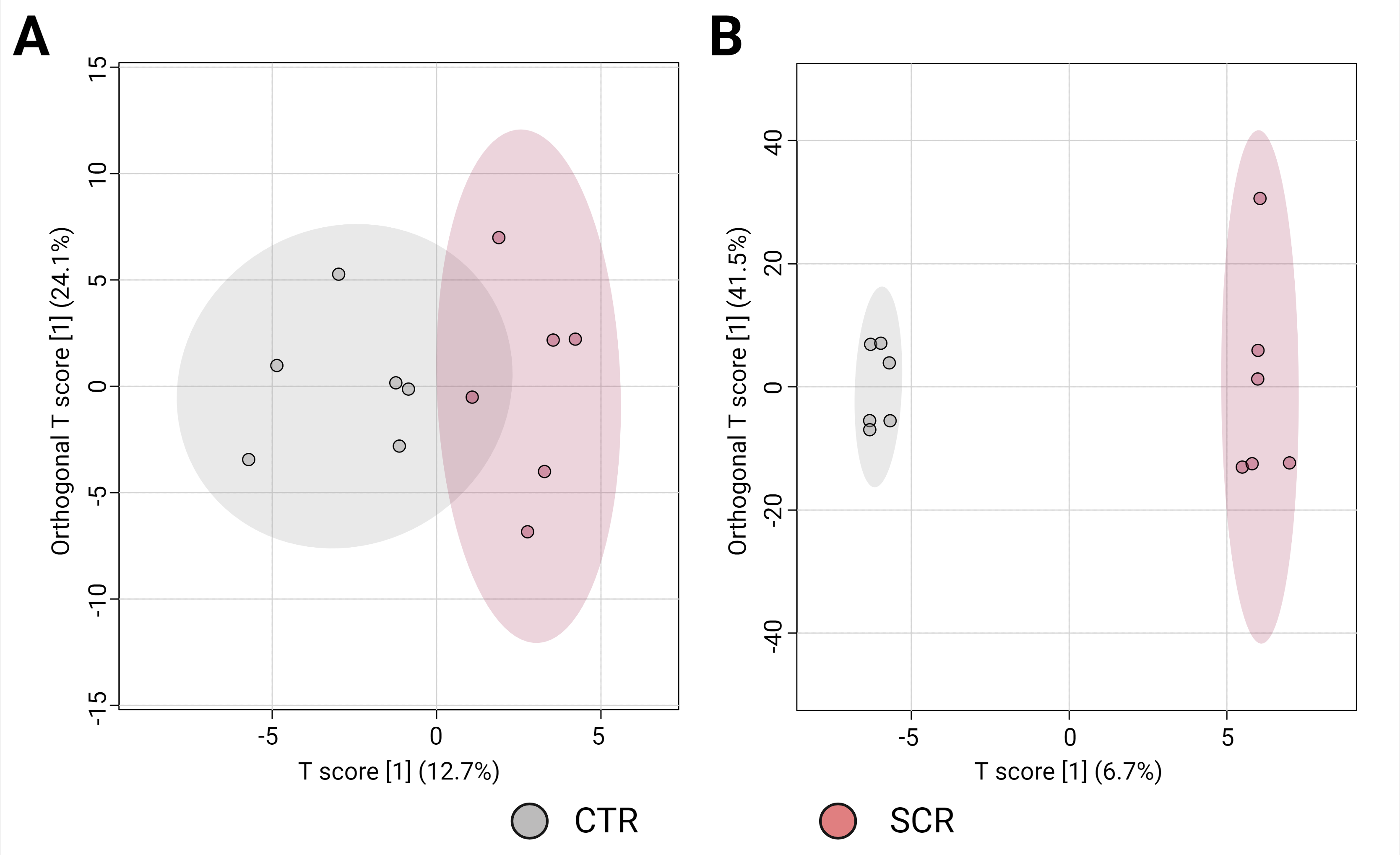

Supplement: Supplementary file 3 — Supplementary Material 3: Fig S3. Multivariate OPLS-DA for the comparison between the CTR and SCR groups. A GC/MS, R²: 0.76, Q²: 0.242, cv-ANOVA: 0.7. B HILIC/MS (-), R²: 0.688, Q²: -0.065, cv-ANOVA: 1. Gray and red dots represent the CTR and scrambled CZS-5, respectively. [file 13071_2025_6861_MOESM3_ESM.tif]
