# Supplementary material for: Exploring the mechanisms of action of the antimicrobial peptide CZS-5 against Trypanosoma cruzi epimastigotes: insights from metabolomics and molecular dynamics
Source: Parasit Vectors. 2025 Jun 5;18:208. doi: 10.1186/s13071-025-06861-5 (PMC12139317; doi:10.1186/s13071-025-06861-5)
Supplement: Supplementary file 5 — Supplementary Material 5: Table S1. Altered metabolites between CTR and those treated with the peptide CZS-5 at 2 and 4 h. [file 13071_2025_6861_MOESM5_ESM.docx]

**Table S1.** Altered metabolites between untreated parasites (CTR) and those treated with the peptide CZS-5 at 2 and 4 hours

| **Name** | **Formula** | **Mass** | **RT** | **mz Error (ppm)** | **Adduct** | **^a^%CV QC** | **A.P.** | **^b^ID level** | **^c^F.C CZS52H VS CTR** | **^c^F.C CZS54H VS CTR** | **^c^F.C CZS52H VS CZS54H** | **^d^P value (FDR)** | **Comp.** | **^e^VIP** |
| --- | --- | --- | --- | --- | --- | --- | --- | --- | --- | --- | --- | --- | --- | --- |
| ***Amino acids, peptides and derivatives*** | | | | | | | | | | | | | | |
| Alanine | C_3_H_7_NO_2_ | 89.0477 | 11.8 | -- | -- | 7.48 | GC/MS | 2 | 0.45 | 0.29 | 1.55 | 8.0E-3 | CTR - CZS52H; CTR - CZS54H | 1.8 |
| Aminolevulinic acid | C_5_H_9_NO_3_ | 131.0583 | 7.8 | 4 | [M-H] ^−^ | 2.68 | HILIC/MS | 2 | 0.47 | 0.36 | 1.33 | -- | -- | 1.4 |
| Aspartic acid | C_4_H_7_NO_4_ | 133.0375 | 11.7 | -- | -- | 18.72 | GC/MS | 2 | 5.76 | 1.82 | 3.15 | 3.6E-2 | CZS52H - CTR; CZS52H - CZS54H | -- |
| Dipeptide | C_8_H_14_N_2_O_5_ | 218.0903 | 8.8 | 2 | [M-H] ^−^ | 3.11 | HILIC/MS | 2 | 0.73 | 1.13 | 0.65 | 3.3E-2 | CTR - CZS52H; CZS54H - CZS52H | -- |
| Glutamic acid | C_5_H_9_NO_4_ | 147.0532 | 14.1 | -- | -- | 5.31 | GC/MS | 2 | 1.63 | 1.32 | 1.23 | 1.0E-2 | CZS52H - CTR; CZS54H - CTR; CZS52H - CZS54H | -- |
| Glutamylglycine | C_7_H_12_N_2_O_5_ | 204.0747 | 9.2 | 2 | [M-H] ^−^ | 6.18 | HILIC/MS | 2 | 0.42 | 0.55 | 0.76 | 7.0E-5 | CTR - CZS52H; CTR - CZS54H | 1.7 |
| Glutathione | C_10_H_17_N_3_O_6_S | 307.0835 | 8.9 | 3 | [M-H] ^−^ | 5.36 | HILIC/MS | 2 | 1.10 | 1.49 | 0.74 | 1.0E-2 | CZS54H - CTR; CZS54H - CZS52H | 1.9 |
| Leucine | C_6_H_13_NO_2_ | 131.0946 | 9.7 | -- | -- | 3.74 | GC/MS | 2 | 0.60 | 1.00 | 0.59 | 4.5E-2 | CTR - CZS52H; CZS54H - CZS52H | -- |
| Methylalanine | C_4_H_9_NO_2_ | 103.0633 | 8.2 | -- | -- | 10.45 | GC/MS | 2 | 0.58 | 0.55 | 1.07 | 2.9E-3 | CTR - CZS52H; CTR - CZS54H | 1.8 |
| Pipecolic acid | C_6_H_11_NO_2_ | 129.0789 | 10.9 | -- | -- | 7.31 | GC/MS | 2 | 0.62 | 0.71 | 0.88 | 1.7E-2 | CTR - CZS52H; CTR - CZS54H | 1.3 |
| Proline | C_5_H_9_NO_2_ | 115.0633 | 10.0 | -- | -- | 4.97 | GC/MS | 2 | 0.50 | 0.63 | 0.79 | 3.0E-2 | CTR - CZS52H; CTR - CZS54H | 1.2 |
| Serine | C_3_H_7_NO_3_ | 105.0425 | 10.9 | -- | -- | 4.53 | GC/MS | 2 | 0.73 | 1.06 | 0.69 | 3.6E-2 | CTR - CZS52H; CZS54H - CZS52H | -- |
| Threonine | C_4_H_9_NO_3_ | 119.0582 | 11.3 | -- | -- | 3.10 | GC/MS | 2 | 0.63 | 1.05 | 0.60 | 3.6E-2 | CTR - CZS52H; CZS54H - CZS52H | -- |
| Tripeptide | C_11_H_19_N_3_O_6_ | 289.1274 | 8.8 | 2 | [M-H] ^−^ | 4.40 | HILIC/MS | 3 | 0.51 | 0.44 | 1.16 | 7.9E-5 | CTR - CZS52H; CTR - CZS54H | 2.1 |
| Tripeptide 2 | C_19_H_25_N_3_O_4_ | 359.1847 | 6.6 | 1 | [M-H] ^−^ | 10.41 | HILIC/MS | 2 | 0.30 | 0.25 | 1.22 | 3.4E-2 | CTR - CZS52H; CTR - CZS54H | 1.6 |
| Tripeptide 3 | C_16_H_23_N_3_O_5_S | 405.1114 | 8.7 | 4 | [M+Cl] ^−^ | 6.14 | HILIC/MS | 4 | 2.77 | 3.03 | 0.92 | 7.9E-5 | CZS52H - CTR; CZS54H - CTR | 2.1 |
| Tryptophan | C_11_H_12_N_2_O_2_ | 204.0898 | 19.9 | -- | -- | 5.48 | GC/MS | 2 | 0.57 | 0.70 | 0.82 | 3.0E-2 | CTR - CZS52H; CTR - CZS54H | 1.1 |
| ***Carbohydrates and carbohydrate conjugates*** | | | | | | | | | | | | | | |
| Anhydro-sorbitol | C_6_H_12_O_5_ | 164.0684 | 16.5 | -- | -- | 3.02 | GC/MS | 2 | 1.42 | 1.40 | 1.01 | -- | -- | 1.1 |
| Arabitol | C_5_H_12_O_5_ | 152.0684 | 15.5 | -- | -- | 7.17 | GC/MS | 2 | 0.82 | 0.89 | 0.92 | 1.5E-2 | CTR - CZS52H; CTR - CZS54H | 1.0 |
| Carbohydrate (C_12_H_22_O_11_) | C_12_H_22_O_11_ | 378.0923 | 8.7 | 3 | [M+Cl] ^−^ | 4.49 | HILIC/MS | 3 | 2.66 | 2.83 | 0.94 | 1.4E-5 | CZS52H - CTR; CZS54H - CTR | 2.2 |
| Galacturonic acid | C_6_H_10_O_7_ | 176.0311 | 1.7 | 5 | [M-H-H_2_O] ^−^ | 10.77 | HILIC/MS | 2 | 0.52 | 0.66 | 0.79 | -- | -- | 1.1 |
| Glucose 6-phosphate | C_6_H_13_O_9_P | 260.0296 | 9.6 | 2 | [M-H] ^−^ | 2.47 | HILIC/MS | 2 | 1.44 | 1.58 | 0.91 | -- | -- | 1.4 |
| Glyceric acid | C_3_H_6_O_4_ | 106.0266 | 10.5 | -- | -- | 3.17 | GC/MS | 2 | 1.29 | 1.33 | 0.97 | -- | -- | 1.0 |
| Glycerol | C_3_H_8_O_3_ | 92.0473 | 9.7 | -- | -- | 5.61 | GC/MS | 2 | 1.63 | 1.47 | 1.11 | 1.7E-3 | CZS52H - CTR; CZS54H - CTR | 1.4 |
| Leucrose | C_12_H_22_O_11_ | 342.1162 | 24.6 | -- | -- | 14.28 | GC/MS | 2 | 4.39 | 3.96 | 1.11 | 1.1E-4 | CZS52H - CTR; CZS54H - CTR | 1.7 |
| Phosphoglycerate | C_3_H_7_O_7_P | 185.9929 | 16.0 | -- | -- | 21.67 | GC/MS | 2 | 1.57 | 1.33 | 1.18 | 3.6E-3 | CZS52H - CTR; CZS54H - CTR; CZS52H - CZS54H | 1.1 |
| Ribitol/Xylitol | C_5_H_12_O_5_ | 152.0684 | 15.3 | -- | -- | 7.79 | GC/MS | 2 | 0.70 | 0.73 | 0.96 | 6.1E-3 | CTR - CZS52H; CTR - CZS54H | 1.6 |
| Ribose | C_5_H_10_O_5_ | 150.0528 | 14.9 | -- | -- | 5.05 | GC/MS | 2 | 2.16 | 2.49 | 0.87 | -- | -- | 1.5 |
| Ribose 5-phosphate | C_5_H_11_O_8_P | 230.0191 | 19.4 |  | [M-H] ^−^ | 9.21 | HILIC/MS-GC/MS | 2 | 1.76 | 2.30 | 0.76 | 6.8E-3 | CZS52H - CTR; CZS54H - CTR | 1.9 |
| Sorbitol/manitol | C_6_H_14_O_6_ | 182.0789 | 7.0 | 4 | [M-H] ^−^ | 5.21 | HILIC/MS | 2 | 0.49 | 0.68 | 0.72 | -- | -- | 1.0 |
| Xylose | C_5_H_10_O_5_ | 150.0528 | 14.0 | -- | -- | 2.54 | GC/MS | 2 | 1.37 | 1.27 | 1.08 | 3.0E-2 | CZS52H - CTR; CZS54H - CTR | 1.2 |
| ***Carboxylic acids and derivatives*** | | | | | | | | | | | | | | |
| Fumaric acid | C_4_H_4_O_4_ | 116.0109 | 10.6 | -- | -- | 7.49 | GC/MS | 2 | 1.05 | 0.76 | 1.39 | -- | -- | 1.0 |
| Phenyllactic acid | C_9_H_10_O_3_ | 166.0631 | 1.3 | 3 | [M-H] ^−^ | 5.37 | HILIC/MS-GC/MS | 2 | 0.54 | 0.63 | 0.87 | 8.4E-3 | CTR - CZS52H; CTR - CZS54H | 1.5 |
| Phosphoenolpyruvic acid | C_3_H_5_O_6_P | 167.9822 | 8.9 | 4 | [M-H] ^−^ | 1.89 | HILIC/MS-GC/MS | 2 | 1.34 | 1.08 | 1.24 | 1.3E-4 | CZS52H - CTR; CZS52H - CZS54H | -- |
| Phosphoric acid | H_3_O_4_P | 97.9768 | 9.7 | -- | -- | 2.19 | GC/MS | 2 | 1.57 | 1.60 | 0.98 | -- | -- | 1.1 |
| Pyruvic acid | C_3_H_4_O_3_ | 88.016 | 6.4 | -- | -- | 2.16 | GC/MS | 2 | 1.71 | 1.19 | 1.44 | 1.7E-3 | CZS52H - CTR; CZS52H - CZS54H | -- |
| Succinic acid | C_4_H_6_O_4_ | 118.0267 | 2.6 | 4 | [M-H] ^−^ | 9.98 | HILIC/MS | 2 | 0.63 | 0.65 | 0.97 | -- | -- | 1.3 |
| Tartronic acid | C_3_H_4_O_5_ | 120.0058 | 10.7 | -- | -- | 4.89 | GC/MS | 2 | 0.65 | 0.67 | 0.96 | 3.6E-2 | CTR - CZS52H; CTR - CZS54H | 1.3 |
| ***Fatty acids and Sterols*** | | | | | | | | | | | | | | |
| Aminoisobutyric acid | C_4_H_9_NO_2_ | 103.0633 | 8.5 | -- | -- | 7.64 | GC/MS | 2 | 0.44 | 0.30 | 1.48 | 3.0E-2 | CTR - CZS52H; CTR - CZS54H | 1.6 |
| CE 15:0 | C_42_H_74_O_2_ | 646.5412 | 5.4 | 8 | [M+Cl] ^−^ | 6.53 | HILIC/MS | 4 | 0.91 | 0.90 | 1.01 | -- | -- | 1.2 |
| Erucamide | C_22_H_43_NO | 337.3344 | 24.7 | -- | -- | 27.97 | GC/MS | 2 | 1.63 | 2.38 | 0.69 | -- | -- | 1.2 |
| FA 22:5 | C_22_H_34_O_2_ | 330.2557 | 0.67 | 2 | [M-H] ^−^ | 3.76 | HILIC/MS | 3 | 1.30 | 1.83 | 0.71 | -- | -- | 1.3 |
| FAL 5:0 | C_5_H_10_O | 86.0733 | 0.82 | 5 | [M-H] ^−^ | 5.52 | HILIC/MS | 3 | 0.35 | 0.60 | 0.59 | 2.4E-3 | CTR - CZS52H; CTR - CZS54H; CZS54H - CZS52H | 1.3 |
| Hydroxybutyric acid | C_4_H_8_O_3_ | 104.0473 | 8.0 | -- | -- | 2.33 | GC/MS | 2 | 0.58 | 0.58 | 1.01 | 1.0E-3 | CTR - CZS52H; CTR - CZS54H | 1.8 |
| Hydroxyisocaproic acid | C_6_H_12_O_3_ | 132.0786 | 9.2 | -- | -- | 5.90 | GC/MS | 2 | 0.43 | 0.68 | 0.64 | 9.0E-3 | CTR - CZS52H; CTR - CZS54H | 1.0 |
| Hydroxyisovalerate | C_5_H_10_O_3_ | 118.0629 | 8.8 |  | -- | 10.57 | GC/MS | 2 | 1.55 | 1.05 | 1.48 | 2.8E-2 | CZS52H - CTR; CZS52H - CZS54H | 0.1 |
| Hydroxy-methylbutyric acid | C_5_H_10_O_3_ | 118.0629 | 8.1 | -- | -- | 3.90 | GC/MS | 2 | 0.44 | 0.42 | 1.03 | 1.0E-3 | CTR - CZS52H; CTR - CZS54H | 1.8 |
| Hydroxy-methylglutaric acid | C_6_H_10_O_5_ | 162.0528 | 13.4 | -- | -- | 4.94 | GC/MS | 2 | 1.08 | 1.26 | 0.86 | -- | -- | 1.0 |
| Hydroxymethylthio butanoic acid | C_5_H_10_O_3_S | 150.035 | 1.6 | 4 | [M-H] ^−^ | 2.43 | HILIC/MS | 2 | 0.36 | 0.49 | 0.72 | 3.2E-2 | CTR - CZS52H; CTR - CZS54H | 1.4 |
| Linoleic acid | C_18_H_32_O_2_ | 280.2402 | 20.0 | -- | -- | 7.26 | GC/MS | 2 | 1.67 | 1.63 | 1.02 | 2.3E-2 | CZS52H - CTR; CZS54H - CTR | 1.4 |
| Methyl Heptadecanoate | C_18_H_36_O_2_ | 284.2715 | 18.0 | -- | -- | 3.28 | GC/MS | 2 | 1.57 | 1.57 | 1.00 | -- | -- | 1.0 |
| Myristic acid | C_14_H_28_O_2_ | 228.2089 | 16.5 | -- | -- | 17.23 | GC/MS | 2 | 1.51 | 1.67 | 0.91 | -- | -- | 1.3 |
| Oleic acid | C_18_H_34_O_2_ | 282.2558 | 20.0 | -- | -- | 6.02 | GC/MS | 2 | 1.62 | 1.57 | 1.03 | 8.1E-3 | CZS52H - CTR; CZS54H - CTR | 1.5 |
| Palmitoleic acid | C_16_H_30_O_2_ | 254.2245 | 18.2 | -- | -- | 5.87 | GC/MS | 2 | 1.65 | 1.79 | 0.92 | 1.9E-2 | CZS52H - CTR; CZS54H - CTR | 1.6 |
| ***Glycerolipids*** | | | | | | | | | | | | | | |
| Monomyristin | C_17_H_34_O_4_ | 302.2457 | 21.6 | -- | -- | 8.18 | GC/MS | 2 | 1.68 | 1.70 | 0.99 | -- | -- | 1.2 |
| Monopalmitin | C_19_H_38_O_4_ | 330.277 | 23.1 | -- | -- | 7.04 | GC/MS | 2 | 1.63 | 1.56 | 1.05 | -- | -- | 1.0 |
| Stearoyl-glycerol | C_21_H_42_O_4_ | 358.3083 | 24.5 | -- | -- | 21.85 | GC/MS | 2 | 1.77 | 1.65 | 1.07 | -- | -- | 1.0 |
| ***Glycerophospholipids*** | | | | | | | | | | | | | | |
| LPC 16:1 | C_24_H_48_NO_7_P | 539.3227 | 5.3 | 0 | [M+HCOOH-H] ^−^ | 5.36 | HILIC/MS | 2 | 1.35 | 1.47 | 0.92 | -- | -- | 1.6 |
| LPC 18:1 | C_26_H_52_NO_7_P | 567.3523 | 5.0 | 4 | [M+HCOOH-H] ^−^ | 4.36 | HILIC/MS | 2 | 1.15 | 1.27 | 0.91 | -- | -- | 1.2 |
| LPC 18:2 | C_26_H_50_NO_7_P | 555.3095 | 5.1 | 1 | [M+Cl] ^−^/[M+HCOOH-H] ^−^ | 5.52 | HILIC/MS | 2 | 1.28 | 1.43 | 0.89 | 1.9E-2 | CZS52H - CTR; CZS54H - CTR | 1.9 |
| LPC 18:2 i2 | C_26_H_50_NO_7_P | 565.3384 | 5.3 | 1 | [M+HCOOH-H] ^−^ | 2.78 | HILIC/MS | 2 | 1.59 | 1.83 | 0.87 | -- | -- | 1.6 |
| LPC 22:6 | C_30_H_50_NO_7_P | 613.338 | 5.2 | 1 | [M+HCOOH-H] ^−^ | 5.56 | HILIC/MS | 3 | 1.57 | 3.63 | 0.43 | -- | -- | 1.1 |
| LPE 18:2 | C_23_H_44_NO_7_P | 477.286 | 5.5 | 0 | [M-H] ^−^ | 6.10 | HILIC/MS | 2 | 1.19 | 1.27 | 0.94 | -- | -- | 1.0 |
| LPE O-16:1 | C_21_H_44_NO_6_P | 437.291 | 5.4 | 0 | [M-H] ^−^ | 5.27 | HILIC/MS | 2 | 1.78 | 1.66 | 1.07 | 4.0E-3 | CZS52H - CTR; CZS54H - CTR | 1.6 |
| LPG22:0 | C_28_H_57_O_9_P | 604.3488 | 5.1 | 4 | [M+Cl] ^−^ | 4.92 | HILIC/MS | 3 | 1.32 | 1.45 | 0.91 | 1.5E-2 | CZS52H - CTR; CZS54H - CTR | 1.9 |
| LPI 18:1 | C_27_H_51_O_12_P | 598.3111 | 6.5 | 2 | [M-H] ^−^ | 2.82 | HILIC/MS | 2 | 1.79 | 1.91 | 0.94 | 3.7E-3 | CZS52H - CTR; CZS54H - CTR | 1.9 |
| LPI18:2 | C_27_H_49_O_12_P | 596.2967 | 6.5 | 0 | [M-H] ^−^ | 3.22 | HILIC/MS | 2 | 1.55 | 1.52 | 1.02 | -- | -- | 1.2 |
| PA 41:6 | C_44_H_75_O_8_P | 762.5156 | 2.9 | 6 | [M-H] ^−^ | 2.22 | HILIC/MS | 4 | 1.21 | 1.27 | 0.95 | -- | -- | 1.2 |
| PA O-42:3 | C_45_H_85_O_7_P | 750.5952 | 0.6 | 3 | [M-H-H_2_O] ^−^ | 2.16 | HILIC/MS | 3 | 0.61 | 0.55 | 1.12 | -- | -- | 1.4 |
| PC 32:3 | C_40_H_74_NO_8_P | 773.5201 | 2.5 | 2 | [M+HCOOH-H] ^−^ | 4.84 | HILIC/MS | 2 | 0.98 | 0.91 | 1.07 | -- | -- | 1.0 |
| PC 35:4 | C_43_H_78_NO_8_P | 813.5484 | 2.3 | 5 | [M+HCOOH-H]^−^ | 2.40 | HILIC/MS | 2 | 1.14 | 1.24 | 0.93 | -- | -- | 1.3 |
| PC 38:7 | C_46_H_78_NO_8_P | 849.5522 | 2.1 | 0 | [M+HCOOH-H]^−^ | 4.89 | HILIC/MS | 2 | 1.23 | 1.52 | 0.81 | -- | -- | 1.3 |
| PC O-31:1/PE O-34:1 | C_39_H_78_NO_7_P | 685.5407 | 3.6 | 0 | [M-H-H_2_O]^−^ | 2.29 | HILIC/MS | 4 | 1.15 | 1.06 | 1.08 | 1.4E-2 | CZS52H - CTR; CZS52H - CZS54H | -- |
| PC O-32:3/PE O-35:3 | C_40_H_76_NO_7_P | 713.5351 | 2.8 | 2 | [M-H]^−^ | 1.91 | HILIC/MS | 4 | 1.12 | 1.15 | 0.97 | -- | -- | 1.2 |
| PE 25:4;O3 | C_30_H_52_NO_11_P | 633.3257 | 5.1 | 4 | [M-H]^−^ | 5.08 | HILIC/MS | 3 | 1.28 | 1.43 | 0.89 | 8.1E-3 | CZS52H - CTR; CZS54H - CTR | 2.0 |
| PE 32:1 | C_37_H_72_NO_8_P | 689.4988 | 3.4 | 2 | [M-H]^−^ | 2.61 | HILIC/MS | 2 | 0.85 | 0.83 | 1.03 | 2.0E-3 | CTR - CZS52H; CTR - CZS54H | 2.0 |
| PE 32:2 | C_37_H_70_NO_8_P | 687.4835 | 3.4 | 1 | [M-H]^−^ | 3.44 | HILIC/MS | 2 | 0.84 | 0.75 | 1.13 | 2.9E-3 | CTR - CZS52H; CTR - CZS54H | 2.1 |
| PE 34:2 | C_39_H_74_NO_8_P | 715.5151 | 3.3 | 1 | [M-H]^−^ | 2.09 | HILIC/MS | 2 | 0.96 | 0.90 | 1.07 | -- | -- | 1.3 |
| PE 34:3 | C_39_H_72_NO_8_P | 713.4989 | 3.3 | 2 | [M-H]^−^ | 2.91 | HILIC/MS | 2 | 0.96 | 0.92 | 1.04 | -- | -- | 1.0 |
| PE 37:3/PC 34:3 | C_42_H_78_NO_8_P | 791.5228 | 2.4 | 1 | [M+Cl]^−^ | 3.76 | HILIC/MS | 4 | 1.25 | 1.47 | 0.85 | -- | -- | 1.0 |
| PE O-32:2 | C_37_H_72_NO_7_P | 673.5041 | 4.1 | 2 | [M-H]^−^ | 1.67 | HILIC/MS | 2 | 0.83 | 0.79 | 1.06 | -- | -- | 1.6 |
| PE O-34:3 | C_39_H_74_NO_7_P | 699.5204 | 2.9 | 1 | [M-H]^−^ | 1.63 | HILIC/MS | 2 | 1.13 | 1.14 | 0.99 | -- | -- | 1.6 |
| PE O-36:5 | C_41_H_74_NO_7_P | 723.5198 | 3.8 | 1 | [M-H]^−^ | 2.47 | HILIC/MS | 2 | 0.91 | 0.83 | 1.11 | -- | -- | 1.1 |
| PG O-38:2 | C_44_H_85_O_9_P | 824.5679 | 5.8 | 3 | [M+Cl]^−^ | 2.88 | HILIC/MS | 4 | 1.56 | 1.36 | 1.14 | 1.3E-4 | CZS52H - CTR; CZS54H - CTR; CZS52H - CZS54H | 1.5 |
| PI 32:2 | C_41_H_75_O_13_P | 806.4945 | 5.8 | 1 | [M-H]^−^ | 4.87 | HILIC/MS | 2 | 0.93 | 0.89 | 1.04 | -- | -- | 1.1 |
| PI 34:1 | C_43_H_81_O_13_P | 836.5419 | 5.8 | 0 | [M-H]^−^ | 2.96 | HILIC/MS | 3 | 1.17 | 1.18 | 0.99 | -- | -- | 1.5 |
| PI 34:3 | C_43_H_77_O_13_P | 832.5107 | 5.8 | 0 | [M-H]^−^ | 3.86 | HILIC/MS | 3 | 1.67 | 1.33 | 1.26 | 7.9E-5 | CZS52H - CTR; CZS54H - CTR; CZS52H - CZS54H | 1.1 |
| PI 35:1 | C_44_H_83_O_13_P | 850.557 | 5.8 | 1 | [M-H]^−^ | 4.20 | HILIC/MS | 2 | 1.16 | 1.26 | 0.92 | -- | -- | 1.4 |
| PI 35:3 | C_44_H_79_O_13_P | 846.526 | 5.9 | 0 | [M-H]^−^ | 2.42 | HILIC/MS | 3 | 1.83 | 1.57 | 1.17 | 7.9E-5 | CZS52H - CTR; CZS54H - CTR; CZS52H - CZS54H | 1.5 |
| PI 36:4 | C_45_H_79_O_13_P | 858.5264 | 5.8 | 0 | [M-H] ^−^ | 3.49 | HILIC/MS | 2 | 1.98 | 1.54 | 1.29 | 1.4E-5 | CZS52H - CTR; CZS54H - CTR; CZS52H - CZS54H | 1.3 |
| PI O-34:3/PG 36:3 | C_43_H_79_O_12_P/C_42_H_77_O_10_P | 818.5313 | 5.7 | 0 | [M-H] ^−^ /[M+HCOOH-H] ^−^ | 4.53 | HILIC/MS | 3 | 1.43 | 1.41 | 1.01 | 2.9E-4 | CZS52H - CTR; CZS54H - CTR | 1.9 |
| PS 34:1/PE 34:2/PC 31:2 | C_40_H_76_NO_10_P/C_39_H_74_NO_8_P | 761.5208 | 5.8 | 1 | [M-H] ^−^/[M+HCOOH-H] ^−^ | 2.73 | HILIC/MS | 3 | 1.20 | 1.26 | 0.95 | -- | -- | 1.2 |
| PS 36:2 | C_42_H_78_NO_10_P | 787.5367 | 5.8 | 0 | [M-H] ^−^ | 4.44 | HILIC/MS | 2 | 1.27 | 1.27 | 1.01 | -- | -- | 1.4 |
| PS 37:1 | C_43_H_82_NO_10_P | 849.5718 | 2.4 | 2 | [M+HCOOH-H] ^−^ | 5.28 | HILIC/MS | 3 | 0.73 | 0.75 | 0.97 | -- | -- | 1.4 |
| PS 37:2 | C_43_H_80_NO_10_P | 847.5569 | 2.5 | 1 | [M+HCOOH-H] ^−^ | 7.20 | HILIC/MS | 3 | 0.71 | 0.73 | 0.97 | 4.3E-2 | CTR - CZS52H; CTR - CZS54H | 1.5 |
| PS 37:6 | C_43_H_72_NO1_0_P | 839.4948 | 6.3 | 1 | [M+HCOOH-H] ^−^ | 18.35 | HILIC/MS | 3 | 1.71 | 1.59 | 1.07 | 8.9E-3 | CZS52H - CTR; CZS54H - CTR | 1.5 |
| PS 38:2/PC 35:3/PE 38:3 | C_44_H_82_NO_10_P/C_43_H_80_NO_8_P | 815.5649 | 2.3 | 4 | [M-H] ^−^/[M+HCOOH-H] ^−^ | 1.87 | HILIC/MS | 4 | 1.14 | 1.26 | 0.91 | -- | -- | 1.2 |
| PS 39:7 | C_45_H_74_NO_10_P | 865.5102 | 6.2 | 1 | [M+HCOOH-H] ^−^ | 12.91 | HILIC/MS | 3 | 0.86 | 0.85 | 1.01 | -- | -- | 1.3 |
| PS 39:8 | C_45_H_72_NO_10_P | 863.4936 | 6.3 | 2 | [M+HCOOH-H] ^−^ | 7.57 | HILIC/MS | 3 | 0.77 | 0.78 | 0.99 | -- | -- | 1.4 |
| [PS 41:2](https://www.lipidmaps.org/resources/tools/chemdb_ontology?abbrev=PS%2041%3A2) | C_47_H_88_NO_10_P | 857.6118 | 2.2 | 4 | [M-H] ^−^/[M+HCOOH-H] ^−^ | 4.23 | HILIC/MS | 4 | 0.82 | 0.78 | 1.05 | -- | -- | 1.6 |
| PS 41:3 | C_47_H_86_NO_10_P | 855.5957 | 2.2 | 4 | [M-H] ^−^ | 2.86 | HILIC/MS | 4 | 0.82 | 0.83 | 0.98 | 2.1E-2 | CTR - CZS52H; CTR - CZS54H | 1.6 |
| PS 41:4 | C_47_H_84_NO_10_P | 853.5819 | 2.2 | 2 | [M-H] ^−^/[M+HCOOH-H] ^−^ | 2.20 | HILIC/MS | 4 | 0.85 | 0.88 | 0.96 | -- | -- | 1.1 |
| PS 41:7 | C_47_H_78_NO_10_P | 883.5065 | 6.2 | 8 | [M+Cl] ^−^ | 6.66 | HILIC/MS | 3 | 1.26 | 1.20 | 1.05 | -- | -- | 1.1 |
| PS 46:5 | C_52_H_92_NO_10_P | 903.6309 | 3.5 | 5 | [M-H-H_2_O] ^−^ | 5.46 | HILIC/MS | 4 | 0.61 | 0.72 | 0.84 | 2.2E-3 | CTR - CZS52H; CTR - CZS54H | 1.5 |
| PS O-36:5/PE O-36:6 | C_42_H_74_NO_9_P/C_41_H_72_NO_7_P | 767.5077 | 2.9 | 4 | [M-H] ^−^ /[M+HCOOH-H] ^−^ | 2.82 | HILIC/MS | 4 | 1.33 | 1.51 | 0.88 | -- | -- | 1.0 |
| ***Nucleosides, nucleotides, and analogues*** | | | | | | | | | | | | | | |
| Adenosine 5'-diphosphate | C_10_H_15_N_5_O_10_P_2_ | 427.201 | 26.6 | -- | -- | 25.21 | GC/MS | 2 | 1.20 | 1.41 | 0.86 | -- | -- | 1.1 |
| Adenosine monophosphate | C_10_H_14_N_5_O_7_P | 347.063 | 8.8 | 2 | [M-H] ^−^/[3M-H] ^−^ | 2.11 | HILIC/MS | 2 | 0.82 | 0.88 | 0.93 | -- | -- | 1.0 |
| Guanosine monophosphate | C_10_H_14_N_5_O_8_P | 399.0329 | 9.2 | 6 | [M+Cl] ^−^ | 3.28 | HILIC/MS | 2 | 1.15 | 1.18 | 0.97 | -- | -- | 1.3 |
| Uridine monophosphate | C_9_H_13_N_2_O_9_P | 324.0358 | 8.8 | 2 | [M-H] ^−^ | 2.40 | HILIC/MS | 2 | 1.05 | 0.73 | 1.45 | -- | -- | 1.1 |
| ***Others*** | | | | | | | | | | | | | | |
| Benzenoid (C_8_H_8_O) | C_8_H_8_O | 120.0575 | 0.97 | 4 | [M-H] ^−^ | 14.55 | HILIC/MS | 3 | 0.6 | 0.7 | 0.9 | 3.4E-2 | CTR - CZS52H; CTR - CZS54H | 1.4 |
| Cadaverine | C_5_H_14_N_2_ | 102.1156 | 16.5 | -- | -- | 5.31 | GC/MS | 2 | 0.4 | 0.4 | 1.0 | -- | -- | 1.3 |
| Ciliatine | C_2_H_8_NO_3_P | 125.0242 | 15.6 | -- | -- | 3.61 | GC/MS | 2 | 0.5 | 0.5 | 1.1 | 1.1E-4 | CTR - CZS52H; CTR - CZS54H | 2.0 |
| Ethanolamine | C_2_H_7_NO | 61.0527 | 9.6 | -- | -- | 7.16 | GC/MS | 2 | 0.7 | 0.9 | 0.8 | 3.0E-2 | CTR - CZS52H; CZS54H - CZS52H | -- |
| Hexadecanoyl sphinganine | C_34_H_69_NO_3_ | 585.534 | 0.67 | 0 | [M+HCOOH-H] ^−^ | 2.17 | HILIC/MS | 2 | 1.1 | 1.1 | 1.0 | -- | -- | 1.0 |
| Indolelactic acid | C_11_H_11_NO_3_ | 205.0737 | 1.49 | 3 | [M-H] ^−^ | 4.27 | HILIC/MS | 2 | 0.5 | 0.6 | 0.8 | 1.0E-2 | CTR - CZS52H; CTR - CZS54H | 1.4 |
| Nicotinamide | C_6_H_6_N_2_O | 122.048 | 12.4 | -- | -- | 6.99 | GC/MS | 2 | 1.0 | 0.7 | 1.3 | -- | -- | 1.2 |
| Triethanolamine | C_6_H_15_NO_3_ | 149.1051 | 14.2 | -- | -- | 4.64 | GC/MS | 2 | 1.7 | 1.9 | 0.9 | -- | -- | 1.05 |
| Xanthine | C_5_H_4_N_4_O_2_ | 152.0332 | 4.5 | 5 | [M-H] ^−^ | 2.08 | HILIC/MS | 2 | 0.9 | 1.2 | 0.7 | -- | -- | 1.2 |

RT: retention time; Comp: Comparison; A.P: Analytical platform; GC: gas chromatography, HILIC: Hydrophilic interaction chromatography. MS: quadrupole time-of-flight mass spectrometer. ^a^CV, coefficient of variation in the metabolites in the QC samples; ^b^Identification level: The lowest level of annotation corresponds to that achieved through exact mass matching (4), followed by molecular formula confirmation (3), identification of specific fragment signals of the compound (2), and confirmation at the standard level (1); ^c^Fold Change, change in the abundance of the specified comparison calculated as (case/control); ^d^p value corresponding to the *p* values calculated by false discovery rate post hoc correction (FDR < 0.05); ^e^VIP, variable importance in projection.
